# Sulfonothioated *meso*-Methyl BODIPY Shows Enhanced Uncaging Efficiency and Releases H_2_S_*n*_

**DOI:** 10.1021/acs.orglett.3c02511

**Published:** 2023-09-05

**Authors:** Lucie Wohlrábová, Jana Okoročenkova, Eduardo Palao, Erika Kužmová, Karel Chalupský, Petr Klán, Tomáš Slanina

**Affiliations:** † Department of Chemistry, Masaryk University, Kamenice 5, 625 00 Brno, Czech Republic; ‡ RECETOX, Masaryk University, Kamenice 5, 625 00 Brno, Czech Republic; § Institute of Organic Chemistry and Biochemistry of the CAS, Flemingovo nám. 542/2, 160 00 Praha 6, Czech Republic; ∥ Institute of Organic Chemistry and Chemical Biology, Goethe University, Max-von-Laue-Str. 7, 60438 Frankfurt am Main, Germany

## Abstract

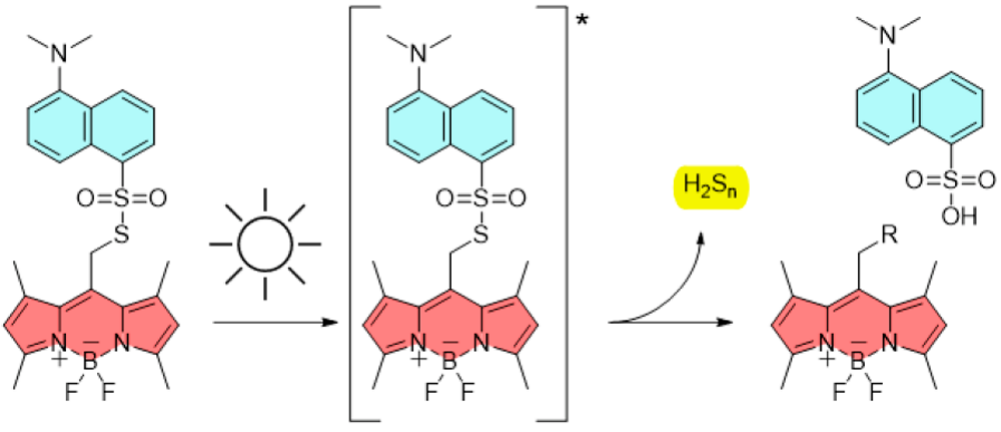

*meso*-Methyl BODIPY photocages stand out for their absorption properties and easy chromophore derivatization. However, their low uncaging efficiencies often hinder applications requiring release of protected substrates in high amounts. In this study, we demonstrate that the sulfonothioated BODIPY group photocleaves a sulfonylthio group from the *meso*-methyl position with a 10-fold higher quantum yield than the most efficient leaving groups studied to date. Photocleavage, observed in solution and in cells, is accompanied by the spatiotemporally controlled photorelease of H_2_S_*n*_. For this reason, sulfonothioated BODIPY may be applied in cell signaling, redox homeostasis, and metabolic regulation studies.

Photoremovable protecting groups (PPGs), also known as photocages, are photosensitive molecules attached to a leaving group (substrate) *via* a covalent bond. The photochemical cleavage of this bond at the desired wavelength enables us to spatiotemporally control the release of the leaving group with high precision.^1^ Such on-demand, on-site substrate release can be used to develop light-responsive compounds for a wide range of applications. These applications depend on the properties of both the substrate and PPG,^1,2^ as shown by the photorelease of signaling lipids,^3^ mitochondrial uncouplers (2,4-dinitrophenol),^4^ or signaling molecules (H_2_S) in living systems.^5−7^

Among PPGs, 4,4-difluoro-4-bora-3a,4a-diaza-s-indacene (BODIPY) stands out for its relatively easy derivatization, low cytotoxicity,^8,9^ and good absorption properties, such as sharp absorption bands, high molar absorption coefficients, and bright emission.^10^ But the photorelease quantum yields of the parent *meso*-methyl BODIPY photocages are usually too low to release sufficient amounts of a substrate.^2,11,12^ Therefore, overcoming this limitation requires an adequate increase of the uncaging efficiency of BODIPY PPGs.

Because BODIPY PPGs efficiently release anions of simple strong acids (e.g., Cl^–^) and their uncaging efficiency increases with the decrease in p*K*_a_ of the leaving group,^2^ we designed and studied a sulfonothioate leaving group with a low p*K*_a_ (≤2).^13^ This sulfonothioate leaving group can be structurally modified and is expected to show high photorelease quantum yields. In addition, this group is a good nucleophile, which enables us to sulfonothioate a BODIPY chromophore through nucleophilic substitution of a halogen atom at the *meso*-methyl position.

In this study, we designed a BODIPY derivative substituted with a thiodansyl, *N*,*N*′-dimethyl-5-[(4,4-difluoro-1,3,5,7-tetramethyl-4*H*-3aλ^4^,4a-diaza-4λ^4^-bora-s-indacen-8-yl)methylthiosulfonyl]-1-naphthylamine **1** as a model molecule (Figure 1). Because the fluorescence properties of thiodansyl differ from those of the BODIPY moiety (λ_em_ = 460 and 530 nm, respectively), we hypothesized that **1** could efficiently release a fluorescent thiodansyl group as a caged fluorophore. Moreover, the photocleavage of a weak S–S bond in the sulfonothioate functional group may enable the release of reactive sulfur species (RSS),^14^ which are relevant in a wide range of cellular mechanisms. Accordingly, sulfonothioated BODIPY may be applied *in vivo* for signaling, redox homeostasis, and metabolic regulation purposes.

**Figure 1:**
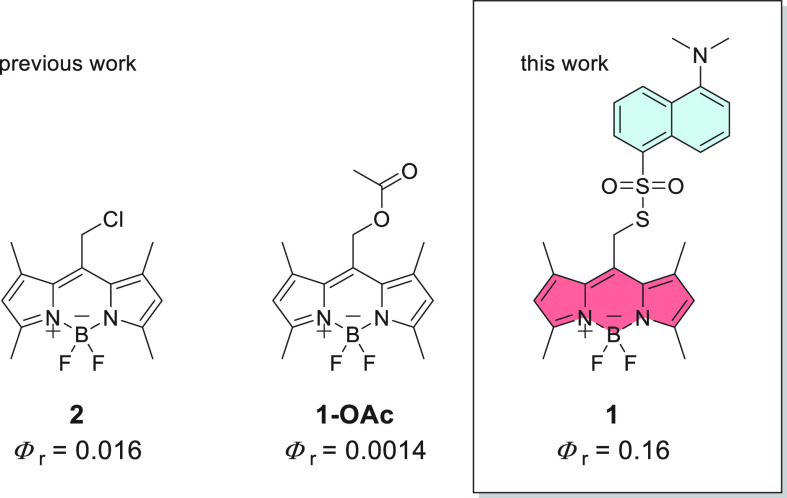
Comparison between the photorelease quantum yields of **1** and the most efficient nonhalogenated *meso-*methyl BODIPY photocages reported in the literature.^2^

We synthesized compound **1** in three steps. In the first step, we prepared BODIPY chloride **2** (Scheme 1). Subsequently, we substituted the chloride for sulfonothioate **3**, previously synthesized in a reaction between dansyl chloride and Na_2_S.

**Scheme 1:**
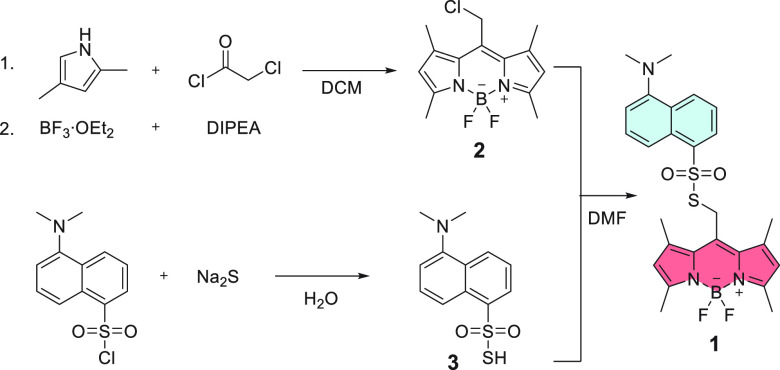
Synthesis of BODIPY Sulfonothioate **1**

Halogen substitution in BODIPY positions 2 and 6 enhances the photorelease quantum yields of common leaving groups from the *meso*-methyl group.^2^ Thus, we synthesized 2,6-halogenated analogs **2-Cl**, **2-Br**, and **2-I** from **2** using the corresponding *N*-halogenosuccinimide. However, the subsequent substitution of the chloride leaving group for **3** unexpectedly yielded sulfinate esters **4-X** (X = Cl, Br, or I), products of formal reduction (Scheme 2). As sulfinates are also good leaving groups, we systematically studied the entire **4-X** series. For this purpose, we further synthesized **4-H** from **2** in a reaction with 5-(dimethylamino)naphthalene-1-sulfinate, resulting from the reduction of **3** with NaHSO_3_.

**Scheme 2:**
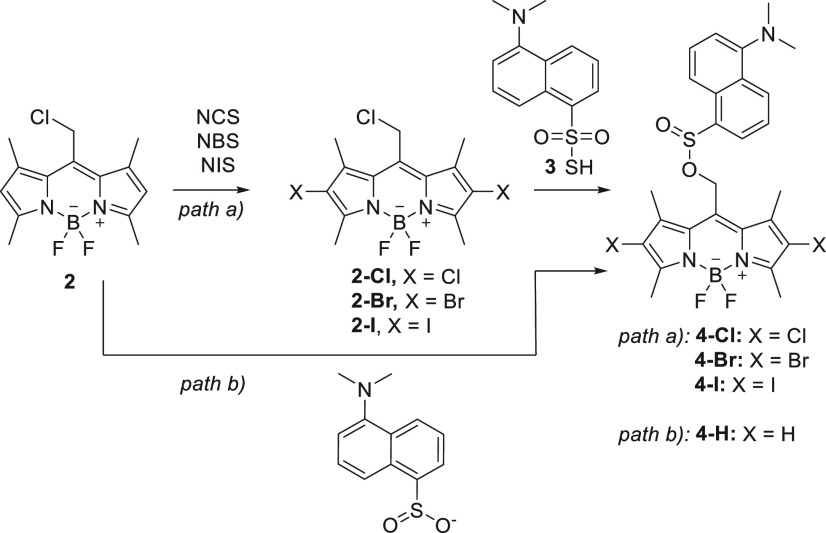
Synthesis of BODIPY Sulfinate Esters **4-X**

The photochemical properties of the target compounds are shown in Table 1. Compound **1** has absorption and emission maxima at 516 and 530 nm (with a Stokes shift Δ*ṽ* of 512 cm^–1^), respectively, and a fluorescence quantum yield Φ_f_ of 0.014. This unusually low fluorescence quantum yield is a consequence of (i) efficient photorelease and (ii) quenching of the excited BODIPY core by charge transfer from the electron-rich sulfur moiety.^15^ Irradiating **1** with green light (λ_irr_ = 525 nm), both in solution and adsorbed on a silica plate soaked with its methanolic solution, yielded highly emissive photoproducts (Figure 2a,b). As a result, a new absorption band appeared at ∼500 nm, and fluorescence was enhanced (up to 7-fold) at ∼530 nm (a BODIPY fragment) and ∼460 nm (a dansyl fragment; Figure 2c,d). The dansyl moieties were released with a quantum yield of 0.16, which is 2–3 orders of magnitude higher than that of acetate released from **1-OAc**.^2^ Photoreactivity was also observed in a DMSO/water mixture (1:1) with an efficiency similar to that in methanolic solutions (Figure S32).

**Table 1:** Spectroscopic and Photochemical Properties of the Synthesized Compounds^a^

	λ_abs_^a^	ε_max_^b^	λ_fluo_^c^	Δ*ṽ*^d^	Φ_f_^e^	Φ_r_^f^
**1**^g^	516	56 000	530	512	0.014 ± 0.002	0.165 ± 0.002^h^
**2**^i^	523	44 400	534	393	0.20 ± 0.002	0.016 ± 0.001
**1-OAc**^i^	517	71 000	529	438	0.73 ± 0.008	0.0014 ± 0.0001

a Absorption maximum in nm.

b Molar absorption coefficient in the absorption maximum in M^–1^ cm^–1^.

c Fluorescence maximum in nm.

d Stokes shift in cm^–1^.

e Fluorescence quantum yield.

f Photorelease quantum yield.

g Measured in aerated dichloromethane/methanol (1:9, v/v) *c* ≈ 2 × 10^–5^ M.

h Determined by irradiation at 525 nm using indolyl fulgide as an actinometer.^16^

i In aerated methanol, *c* ≈ (1–10) × 10^–6^ M (data retrieved from the literature).^2^

**Figure 2:**
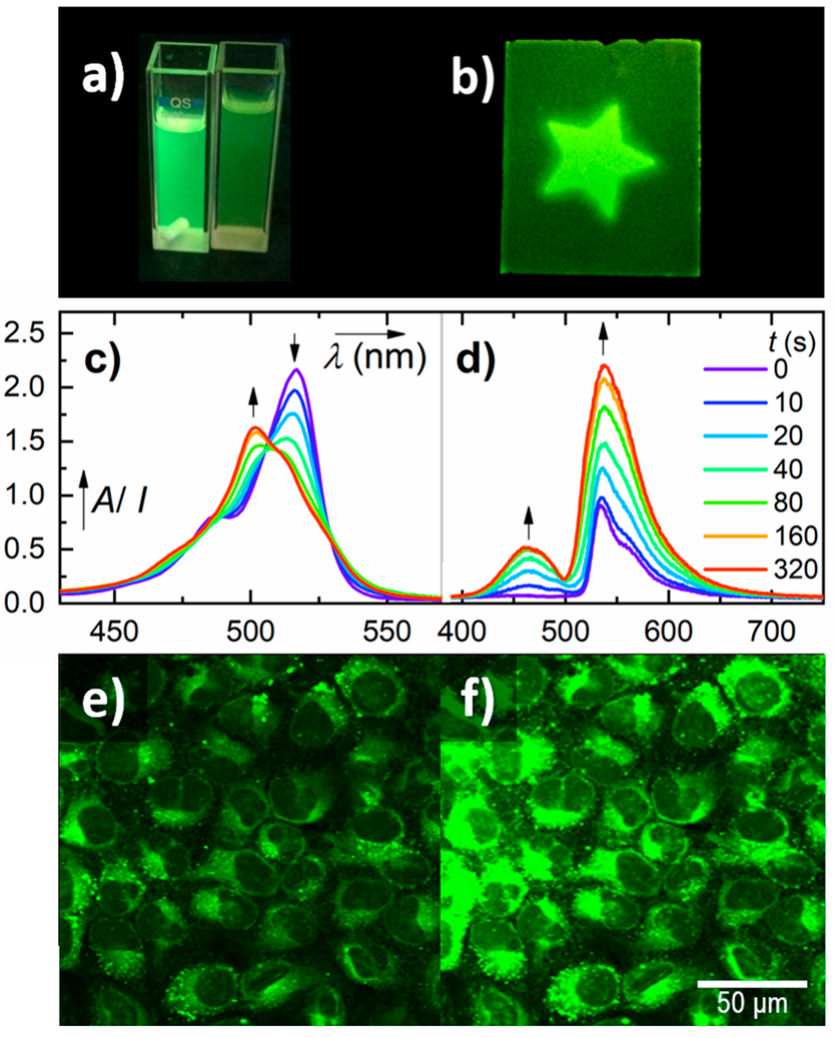
Irradiation of **1** with a 525 nm LED in nondegassed methanol (*c* ∼ 0.1 μM): (a) irradiated (left cuvette) and nonirradiated (right cuvette) solutions under 365 nm light, (b) TLC plate soaked with the solution of **1** immediately irradiated (without drying) through a star-shaped photomask visualized under 365 nm light, and (c) absorption and (d) emission (λ_exc_ = 330 nm) spectra. Fluorescence images of U-2 OS cells treated with **1** (*c* = 100 μM) were acquired after (e) incubation in the dark for 1 min and (f) irradiation with 492 nm light for 3 min. The scale bar is 50 μm.

The absorption properties of sulfinate esters **4** were analogous to those of their acetate counterparts,^2^ but their fluorescence was much weaker (Table S1). Although sulfinate is an excellent leaving group, irradiating BODIPY-sulfinate esters did not release the leaving group, most likely because the excited state was quenched by intramolecular charge transfer from the lone pair of the sulfinate group to the excited BODIPY chromophore.^17^ This process also accounted for their fluorescence quenching.

To demonstrate its potential for biological applications, **1** was administered in U-2 OS cells before monitoring the buildup of fluorescence corresponding to the photorelease of more emissive photoproducts (Figure 2e,f, Figures S34–36). *meso*-Methyl alcohol **5** was released in an aqueous solution, as shown by HPLC-MS analysis (Figure S37). Furthermore, the concentration ratio between the oxidized and reduced forms of glutathione (GSSG/GSH), reflecting redox homeostasis,^18^ increased in the presence of irradiated **1** (Figure S38). Compound **1** was also nontoxic after 24 h at concentrations below 100 μM, which is its solubility limit. Its phototoxicity after 24 h was also minimal (<10 μM, Figure S33).

To understand the photodeprotection mechanism, **1** was irradiated with a 525 nm LED in an aerated dichloromethane/methanol mixture (1:9, v/v). Several products were identified by high-performance liquid chromatography coupled with mass spectrometry detection (HPLC-MS). The main photoproducts were BODIPY *meso*-methyl alcohol **5** and the product of its oxidation, aldehyde **6** (Figure 3a,b). The expected product of photo-S_N_1 solvolysis, methoxy-substituted BODIPY **7**, was detected only in trace amounts (∼7%). These results indicate that the sulfonyl thioate group likely induces significant changes in the photorelease mechanism.

**Figure 3:**
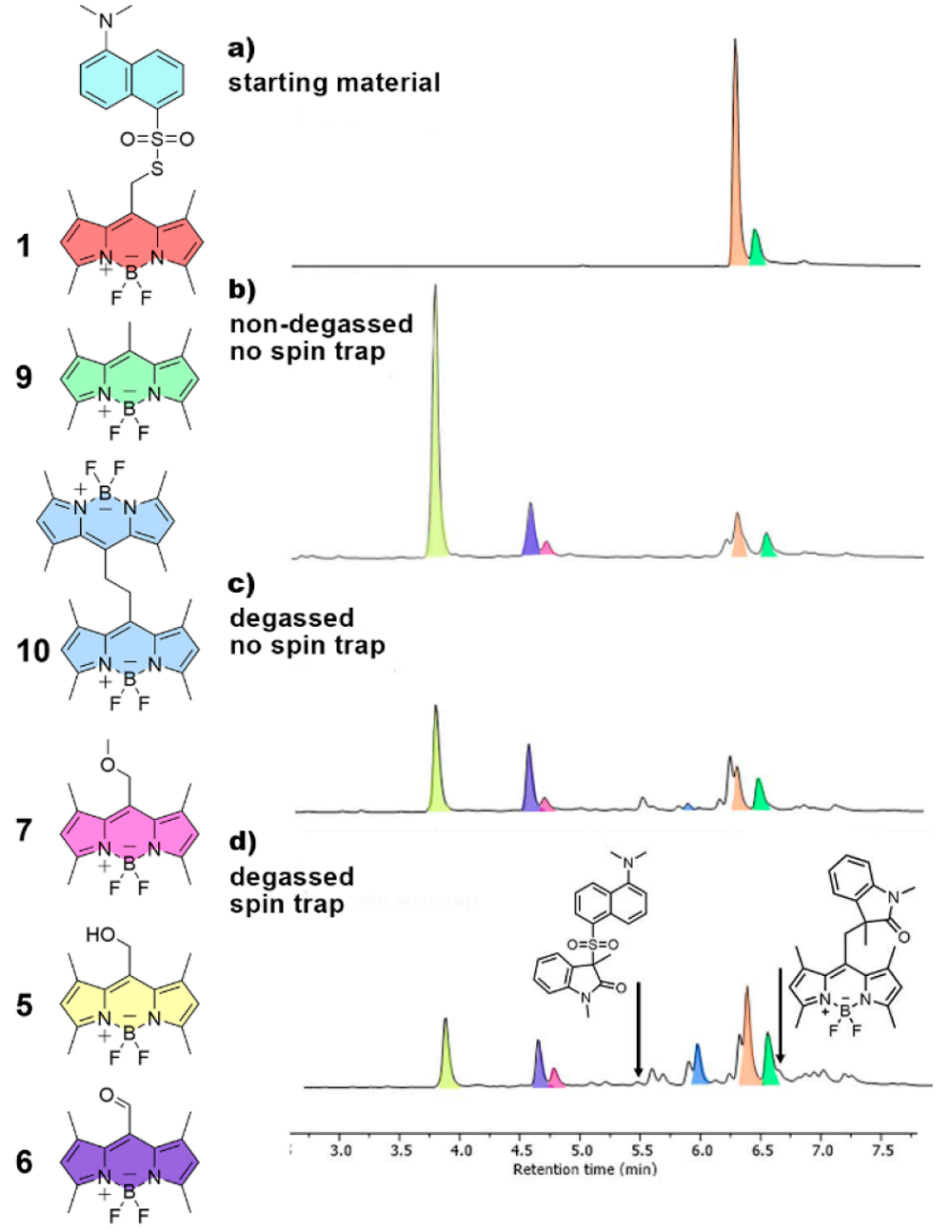
HPLC chromatograms of **1** (a) before and (b) after irradiation with 525 nm LED in nondegassed and (c) degassed solutions. (d) Trapping experiment in an irradiated degassed solution of **1** containing *N*-methyl-*N*-phenyl-methacrylamide as a spin trap.

The amounts of photoproducts **5** and **6** were approximately 2.2 times higher in aerated solutions than in degassed reaction mixtures. Oxygen dissolved in the reaction mixture thus likely played a key role in the reaction mechanism as a radical trap and oxidant. Although **5** and **6** were identified as the two main products in aerated samples, several other compounds were formed in degassed reaction mixtures due to the lack of oxygen, which enabled subsequent reactions of radical intermediates (Figure 3c).

To confirm that **5** was formed in the reaction of a BODIPY *meso*-methyl moiety with oxygen and not with the residual moisture, **1** was irradiated with a 525 nm LED in a degassed dichloromethane/methanol/H_2_^18^O (5:4:1, v/v/v) mixture. High-resolution mass spectrometry analysis did not reveal any product containing isotopically labeled oxygen (Figures S6–9). This fact, together with the oxygen-dependent formation of **5** and **6**, helped us to identify O_2_ as the only source of the hydroxyl oxygen atom in **5**.

We also assessed whether thiodansyl photorelease from **1** proceeded *via* a radical mechanism using a spin trap^19^ (*N*-methyl-*N*-phenyl-methacrylamide, Scheme S2) in degassed and nondegassed solutions by analyzing the resulting photoreaction mixtures by HPLC-MS (Figure 3d). Two 1*-*methylindolin-2-one derivatives were detected in irradiated, degassed mixtures in the presence of a spin trap. However, their accumulation in the reaction mixture was unlikely, given their low thermal and photochemical stability and trapping efficiency and the incomplete deaeration of the irradiated solution (Figure S10). For this reason, we used these results only for qualitative evaluation purposes.

The photochemical cleavage of the sulfonothioate bond in **1** provided dansyl sulfonate **8** (Scheme 3) as the only non-BODIPY-containing chromophoric product. Product **8** was not detected when **1** was heated in the dark (Figure S11). This finding can be explained only by the release of one sulfur atom from the sulfonothioate group during the photochemical process. Supporting this hypothesis, compound **3** photochemically generated **8** upon direct excitation at 365 nm and at 525 nm in the presence of **7** as a sensitizer (Scheme S3). When irradiated alone at 525 nm, compound **3** remained in the solution because it did not absorb in this region.

**Scheme 3:**
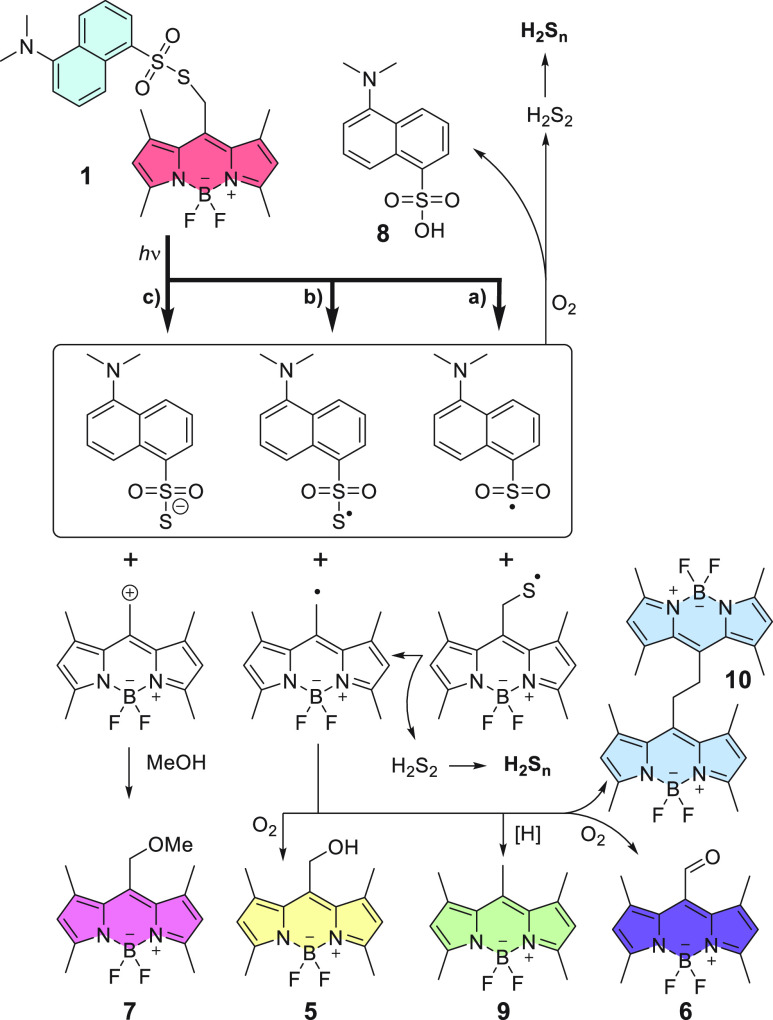
Suggested Mechanism of the Photodegradation of **1**

We also assessed whether H_2_S was released from **1** using the methylene blue assay.^20^ No sign of H_2_S was detected until the addition of glutathione (GSH) to the irradiated mixture (Figure S5). This result indicated the reduction of photochemically generated polysulfides (H_2_S_*n*_).^21^ Using this method, the chemical yield of H_2_S was approximately 76%. To assess whether the polysulfides were produced as H_2_S_2_ or as longer polysulfides (H_2_S_*n*_, *n* > 2), we determined the amount of H_2_S_2_ formed when irradiating **3**. For this purpose, we used a fluorescein probe for H_2_S_2_ (Scheme S3).^22^ The results showed that H_2_S_2_ accounts for 10% yield, while the remaining sulfur-containing products correspond to higher polysulfides.

To determine the multiplicity of the productive excited state in the release, we irradiated compound **1** together with either thioxanthone as a triplet sensitizer or cyclooctatetraene as a triplet quencher (Figures S3–4). The photoreaction was more efficient upon addition of the triplet sensitizer and suppressed when using the triplet quencher. Since the triplet quenching was incomplete, the reaction likely proceeded through a short-lived triplet state or, simultaneously, a singlet excited state.^2^

The suggested reaction mechanism can be summarized as follows. On one hand, photoinduced homolytic cleavage can occur at either *meso*-methyl–S or S–S bonds of **1** (Scheme 3, pathways a and b, respectively, Scheme S4). Both pathways lead to BODIPY *meso*-methylthio or *meso*-methyl radicals. The former releases H_2_S_2_, whereas the latter can (i) abstract hydrogen atoms, forming **9** (photoreduction), (ii) dimerize into **10**, or (iii) form **5** and **6** when trapped by oxygen (O_2_). On the other hand, heterolytic cleavage at the *meso*-methyl position (Scheme 3, pathway c) is a minor (∼7%) pathway, affording **7** as a product of solvolysis and H_2_S_2_ by a sensitized release from **3** (which does not absorb at the wavelength of irradiation). The released H_2_S_2_ is further polymerized in the presence of free radicals.^23^

In conclusion, **1** is a readily synthesized cage compound with a photorelease quantum yield 1 order of magnitude higher than that of the most efficient heavy atom-free *meso*-methyl BODIPY photocages reported so far.^2^ The markedly enhanced deprotection quantum yield of **1** is derived from its dual photodeprotection mechanism: a weak sulfonothioate bond undergoes both heterolytic and homolytic photoinduced cleavage, releasing H_2_S_2_ and other polysulfides. Photorelease from **1** was monitored in U-2 OS cells by fluorescence microscopy. The released reactive sulfur species affected the GSSG/GSH ratio, a redox homeostasis model. Photocage **1** is thus a promising tool for spatiotemporally controlling the release of reactive sulfur species (RSS)^14^ and may be used *in vivo* for studying cell signaling,^24^ redox homeostasis,^25^ metabolic regulation,^26^ and cellular recovery from oxidative stress.^27^ The released polysulfides can also be reductively converted into an important gasotransmitter–hydrogen sulfide.^6,23^

## Data Availability

The data underlying this study are available in the published article and its Supporting Information.

## Supporting Information Available

Materials and methods; synthetic details; NMR, HR-MS, and absorption spectra; H_2_S and H_2_S_2_ determination; spin trapping; hydrolytic stability, D_2_^18^O labeling; triplet sensitization, triplet quenching (PDF)
