# Supplementary material for: Sulfonothioated meso-Methyl BODIPY Shows Enhanced Uncaging Efficiency and Releases H2Sn
Source: Org Lett. 2023 Sep 5;25(36):6705–9. doi: 10.1021/acs.orglett.3c02511 (PMC10510718; doi:10.1021/acs.orglett.3c02511)
Supplement: Supplementary file 1 — ol3c02511_si_001.pdf [file ol3c02511_si_001.pdf]

Supporting Information

Sulfonothioated *meso*-methyl BODIPY shows enhanced uncaging efficiency and releases H_2S_n

Lucie Wohlrábová,[†] Jana Okoročenkova,[‡,§] Eduardo Palao,[‡,§] Erika Kužmová,[†] Karel Chalupský,[†] Petr Klán[*‡,§] and Tomáš Slanina[*†,‖]

[†]Institute of Organic Chemistry and Biochemistry of the CAS, Flemingovo nám. 542/2, 160 00 Praha 6, Czech Republic

[‡]Department of Chemistry, Masaryk University, Kamenice 5, 625 00 Brno, Czech Republic

[§]RECETOX, Masaryk University, Kamenice 5, 625 00 Brno, Czech Republic

[‖]Institute of Organic Chemistry and Chemical Biology, Goethe University, Max-von-Laue-Str. 7, 60438 Frankfurt am Main, Germany

Table of Contents

List of abbreviations 2

Compound characterization 2

Photophysical and photochemical properties of BODIPY-sulfinates 8

Fluorescence quantum yields 9

Photoreaction quantum yields 10

Thioxanthone (TX) sensitization 11

Cyclooctatetraene (COT) quenching 12

H_2S yield determination 13

Sulfonothioate sensitization 14

Labeling by $D_2{}^{18}O$ 15

Spin trapping 18

Photoreactivity and hydrolytic stability of 3 20

Determination of the H_2S_2 yield 21

^{1}H NMR, ^{13}C NMR, and HR-MS spectra 22

Absorption and emission spectra 37

Reactivity in DMSO:water mixture 39

Cell viability assay 40

Confocal microscopy 40
HPLC-MS analysis 42
Suggested mechanism 44
References 45

Experimental section

The reagents and solvents used in the study were purchased from commercial suppliers and used without purification unless stated otherwise. The solvents were removed on a rotary evaporator at 25–40 °C, and the products were dried in vacuo overnight. TLC analysis was performed on silica gel-coated aluminum plates (60F254, Merck, Darmstadt, Germany). The compounds were visualized using one of the following methods: exposure to UV light at 254 nm or 365 nm, $KMnO_4$ spraying (1 % aqueous solution, yellow color of oxidizable compounds), and exposure to iodine vapors (yellow to brown spots, non-specific). Flash chromatography purifications were performed on silica gel (40–63 µm, Sigma-Aldrich). Gradient chromatography was performed on an ECOM flash chromatograph. ^{1}H and ^{13}C NMR spectra were measured on Bruker-400 AVANCE III HD (^{1}H at 400.13 MHz, ^{13}C at 100.62 MHz, ^{19}F at 376 MHz) in $CDCl_3$, CD_3OD or DMSO-d_6 solution at 300 K. H,H-COSY, H,C-HSQC, and H,C-HMBC spectra were recorded and used for the structural assignment of proton and carbon signals. HR-MS (high-resolution mass spectrometry) spectra were recorded on an FTMS mass spectrometer LTQ-orbitrap XL (Thermo Fisher Scientific) in electrospray ionization mode. HPLC-MS was performed on an HPLC-MS-2020 (Shimadzu Corporation). UV vis spectrometry was measured on an Agilent Cary 8454 UV-vis spectrometer (Agilent), and emission spectra were measured on a Duetta fluorescence spectrometer (Horiba). Mixtures of solvents are given in a v/v ratio.

List of abbreviations

CHEX = cyclohexane, DCM = dichloromethane, MeCN = acetonitrile, EtOAc = ethylacetate, TEA = triethylamine, DIPES *N,N*-diisopropylethylamine, TX = thioxanthone, COT = cyclooctatetraene m.p. = melting point, HR-MS = high resolution mass spectrometry, LR-MS = low resolution mass spectrometry

Compound characterization

Sodium 5-(dimethylamino)naphthalene-1-sulfonothioate (3)

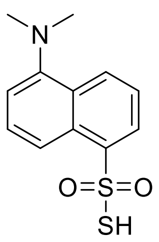

Sodium sulfide (1.48 g, 19.0 mmol, 10 eq) was dissolved in water (20 mL), followed by dansyl chloride addition (0.5 g, 1.9 mmol, 1 eq). The thoroughly stirred suspension was heated to 60 °C for 30 min in a heating mantle. Then, the reaction mixture was brought back to room temperature and stirred for 14 h. Subsequently, the mixture was concentrated to 2 mL, liquid-loaded on a reverse column, and purified by gradient chromatography using a water-MeCN mobile phase modified with 0.05 % TEA (0-50 % of MeCN in water). The solvent was removed, yielding a white solid (490 mg, 30 %).

^{1}H NMR (400.13 MHz, DMSO-*d*6) δ (ppm): 8.83 (1H, dd, J_{HH} = 8.7, 10.0 Hz), 8.21 (1H, dd, J_{HH} = 8.4, 1.1 Hz), 7.99 (1H, dd, J_{HH} = 7.2, 1.3 Hz), 7.44 (2H, dd, J_{HH} = 8.7; 7.2 Hz), 7.14 (1H, dd, J_{HH} = 7.2, 1.0 Hz), 2.81 (2H, s). ^{1}H NMR spectrum corresponds to the literature.[1]

5-(Dimethylamino)naphthalene-1-sulfinic acid

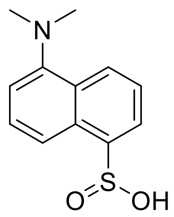

Dansyl chloride (400 mg, 1.5 mmol, 1 eq) was added to a solution of $NaHSO_3$ (154 mg, 1.5 mmol, 1 eq) and K_2CO_3 (204 mg, 1.5 mmol, 1 eq) in water. The suspension was stirred at 50 °C for 8 h in a heating mantle and then stirred at room temperature overnight. Subsequently, the solvent was removed on a rotary evaporator, and the crude product was used in the next reaction without further purification. The purity of the target product was confirmed by HPLC-MS.

8-(Chloromethyl)-4,4-difluoro-1,3,5,7-tetramethyl-4*H* -3aλ^4,4a-diaza- 4λ^4-bora-s-indacene (2)

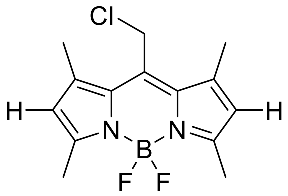

A solution of chloroacetyl chloride (0.78 mL, 9.8 mmol, 1.0 eq) in DCM (15.0 mL) was slowly added to a solution of 2,4-dimethyl-1*H* -pyrrole (2.3 mL, 22.5 mmol, 2.3 eq) in DCM (15 mL) under argon atmosphere. The flask was covered with aluminum foil and heated up to 50 °C in a heating mantle. DIPEA (6.8 mL, 39.2 mmol, 4.0 eq) was subsequently added (cooling to 0 °C), followed by BF_3 etherate (7.3 mL, 58.8 mmol, 6.0 eq). The reaction mixture was stirred for 2 h, and the solution was filtered on SiO_2 and washed with DCM:CHEX (1:1). The filtrate was evaporated to dryness, giving orange powder purified by gradient chromatography using CHEX:DCM (0–100 % DCM) as a mobile phase. The solvent was removed, yielding an orange powder (1 g, 36 %).
^{1}H NMR (400.13 MHz, $CDCl_3$) δ (ppm): 6.09 (2H, s), 4.70 (2H, s), 2.49 (6H, s), 2.47 (6H, s).
^{1}H NMR spectrum corresponds to the literature.[2]

2,6-Dichloro-8-(chloromethyl)-4,4-difluoro-1,3,5,7-tetramethyl-4*H*-3aλ^4,4a-diaza-4λ^4-bora-s-inda-cene (2-Cl)

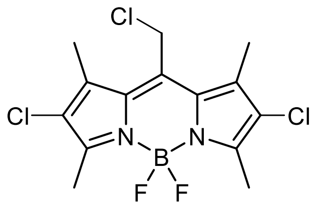

This compound was synthesized according to the literature.[2] A solution of *N*-chlorosuccinimide (297 mg, 2.2 mmol, 2.2 eq) in dry DCM was added to a solution of **2** (300 mg, 1.0 mmol, 1.0 eq) in dry DCM at room temperature under nitrogen atmosphere. The mixture was stirred overnight and then washed with H_2O (3x). The organic layer was evaporated with SiO_2 and dry-loaded on a chromatography column. The crude product was purified by gradient chromatography using CHEX:DCM (0–100 % of DCM) as a mobile phase. The solvent was removed, yielding orange crystals (100 mg, 27 % yield).
^{1}H NMR (400.13 MHz, $CDCl_3$) δ (ppm): 4.77 (2H, s), 2.57 (6H, s), 2.54 (6H, s).
^{1}H NMR spectrum corresponds to the literature.[2]

2,6-Dibromo-8-(chloromethyl)-4,4-difluoro-1,3,5,7-tetramethyl-4*H*-3aλ^4,4a-diaza-4λ^4-bora-s-indacene (2-Br)

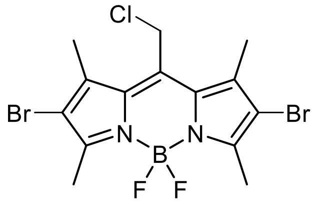

This compound was synthesized according to the literature.[2] A solution of *N* -bromosuccinimide (396 mg, 2.2 mmol, 2.2 eq) in dry DCM was added to a solution of **2** (300 mg, 1.0 mmol, 1.0 eq) in dry DCM under nitrogen atmosphere. The mixture was stirred overnight and washed with H_2O (3x). The organic layer was evaporated with SiO_2 and dry-loaded on the chromatography column. The product was purified by gradient

chromatography using CHEX:DCM (0–100 % of DCM) as a mobile phase. The solvent was removed, yielding dark brown crystals (347 mg, 68 %).

^{1}H NMR (400.13 MHz, $CDCl_3$) δ (ppm): 4.81 (2H, s), 2.63 (6H, s), 2.59 (6H, s).

^{1}H NMR spectrum corresponds to the literature.[2]

2,6-Diiodo-8-(chloromethyl)-4,4-difluoro-1,3,5,7-tetramethyl-4*H*-3aλ^4,4adiaza-4λ^4-bora-s-indacene (2-I)

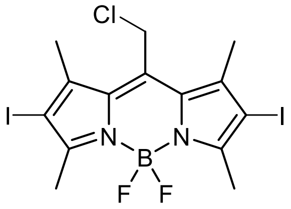

BODIPY **2** (300 mg, 1 mmol, 1.0 eq) was dissolved in DCM (5 mL), subsequently adding a DCM solution of iodine monochloride (328 mg, 2 mmol, 2.0 eq). The reaction mixture was stirred overnight. The formation of the product was monitored by TLC. The solvent was removed on a rotary evaporator with SiO_2 and dry-loaded on the chromatography column. The crude product was purified by gradient chromatography with a CHEX:DCM (0–100 % of DCM) mobile phase, yielding dark brown crystals (290 mg, 59 % yield).

^{1}H NMR (400.13 MHz, $CDCl_3$) δ (ppm): 4.79 (2H, s), 2.59 (6H, s), 2.56 (6H, s).

^{1}H NMR spectrum corresponds to the literature.[2]

***N*,*N'*-Dimethyl-5-[(4,4-difluoro-1,3,5,7-tetramethyl-4*H*-3aλ^4,4a-diaza-4λ^4-bora-s-indacen-8-yl)methylthiosulfonyl]-1-naphthylamine (1)**

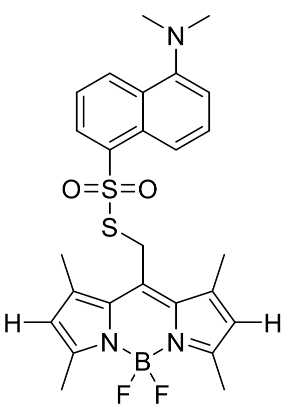

BODIPY **2** (50 mg, 169 µmol, 1 eq) was dissolved in anhydrous acetone, followed by KI addition (45 mg, 270 µmol, 1.6 eq). After 10 minutes of stirring, acetone (3 mL) was removed, and the resulting solid was dissolved in a minimum amount of DMF (3 mL). Compound **3** (67 mg, 253 µmol, 1.5 eq) was dissolved in water, and the pH was adjusted to 7 by titration with NaOH to form a sodium salt. The solvent was removed, and the solid was used for the reaction with **2-Cl** monitored with TLC. Full conversion was observed after 5 minutes of stirring at room temperature. The solvent was removed, and the crude product was purified by gradient chromatography with a DCM:CHEX mobile phase (0–100 % of DCM). The solvent was removed, yielding the target compound as orange-red crystals (48 mg, 54 %).

^{1}H NMR (400.13 MHz, $CDCl_3$) δ (ppm): 8.66 (1H, dd, J_{HH} = 8.5; 1.2 Hz), 8.42 (^{1}H, dd, J_{HH} = 8.7, 1 Hz), 8.30 (^{1}H, dd, J_{HH} = 7.4, 1.31 Hz), 7.62 (2H, m), 7.25 (1H, m), 5.92 (2H, s), 4.27 (2H, s), 2.95 (6H, s), 2.44 (6H, s), 1.90 (6H, s). **^{13}C NMR** (100.62 MHz, $CDCl_3$) δ (ppm): 156.7, 145.8, 141.4, 140.9, 131.7, 130.1, 127.5, 122.5, 39.0, 33.0, 21.9, 14.8. **^{19}F NMR** (376 MHz, $CDCl_3$) δ (ppm): -146.81 (dt, J = 66.4, 34.1 Hz). **HR-MS** (ESI+) m/z calcd. for $C_{26}H_{28}O_2N_3BF_2S_2$ $[M+H]^+$ 528.1757; found 528.1753. **m. p.** = 208–213 °C.

The reaction was also performed on a large scale (1.4 mmol) with 45% yield (730 mg).

_N_,_N'_-Dimethyl-5-[(4,4-difluoro-1,3,5,7-tetramethyl-4_H_-3aλ^4,4a-diaza-4λ^4-bora-s-indacen-8-yl)methoxysulfinyl]-1-naphthylamine (4-H)

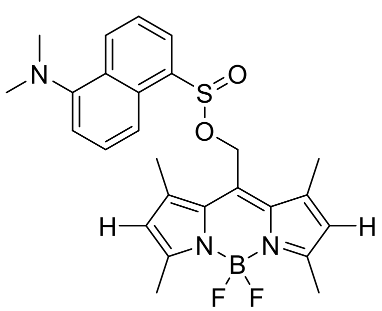

BODIPY **2** (50 mg, 169 µmol, 1 eq) and 5-(dimethylamino)naphthalene-1-sulfinic acid (39 mg, 169 µmol, 1 eq) were dissolved in DMF (2 mL). The reaction mixture was left to stir for 10 minutes. The solvent was removed, and the mixture was dissolved in DCM and co-evaporated with SiO_2. Then, the mixture was dry-loaded on the column, and the crude product was purified by gradient chromatography with a DCM:CHEX mobile phase (0–100 % of DCM). The target compound was obtained as an orange-red solid (40 mg, 48 %).

^{1}H NMR (400.13 MHz, $CDCl_3$) δ (ppm): 8.57 (1H, dd, J_{HH} = 8.5, 1.1 Hz), 8.28 (1H, dd, J_{HH} = 7.4, 1.3 Hz), 8.23 (1H, dd, J_{HH} = 8.8, 1.0 Hz), 7.51 (1H, dd, J_{HH} = 8.5, 7.4 Hz), 7.44 (1H, dd, J_{HH} = 8.7, 7.6 Hz), 7.09 (1H, dd, J_{HH} = 7.6, 1.0), 5.77 (2H, s), 4.95 (2H, s), 2.84 (6H, s), 2.45 (6H, s), 2.05 (6H, s). **^{13}C NMR** (100.62 MHz, $CDCl_3$) δ (ppm): 151.3, 141.6, 134.3, 133.2, 132.3, 131.5, 131.2, 129.5, 129.0, 128.6, 123.3, 122.7, 119.0, 115.2, 56.4, 45.5, 29.9, 16.4, 14.7. **^{19}F NMR** (376 MHz, $CDCl_3$) δ (ppm): -146.88 (ddd, J = 64.6, 31.9, 8.4 Hz). **HR-MS** (ESI-) m/z calcd. for $C_{26}H_{28}O_2N_3BF_2S$ $[M-H]^-$ 494.1891; found 494.1890. **m. p.** = 210–215 °C.

_N_,_N'_-Dimethyl-5-[(2,6-dichloro-4,4-difluoro-1,3,5,7-tetramethyl-4_H_-3aλ^4,4a-diaza-4λ^4-bora-s-indacen-8- l)methoxysulfinyl]-1-naphthylamine (4-Cl)

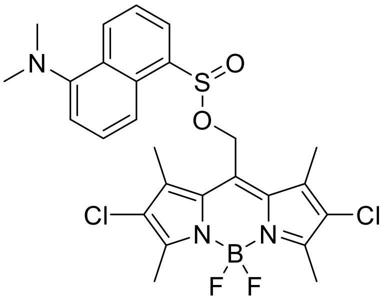

BODIPY **2-Cl** (50 mg, 136 µmol, 1 eq) was dissolved in anhydrous acetone, followed by KI addition (23 mg, 136 µmol, 1.6 eq). After 10 minutes of stirring, acetone (3 mL) was removed, and the resulting solid was dissolved in a minimum amount of DMF (3 mL). Compound **3** (73 mg, 136 µmol, 1.5 eq) was dissolved in water, and the pH was adjusted to 7 by titration with NaOH to form a sodium salt. The solvent was removed, and the solid was used in the reaction with **2-Cl** monitored by TLC. Full conversion was observed after 5 minutes of stirring. Then, the solvent was removed, and the crude product was purified by gradient chromatography using a DCM:CHEX mobile phase (0–100 % DCM in CHEX). The solvent was removed, yielding the target compound as orange-red crystals (10 mg, 13 %).

^{1}H NMR (400.13 MHz, $CDCl_3$) δ (ppm): 8.63 (1H, dd, J_{HH} = 7.4, 1.2 Hz), 8.33 (1H, dd, J_{HH} = 7.4, 1.2 Hz), 8.18 (1H, dd, J_{HH} = 8.8, 1.0 Hz), 7.57 (1H, dd, J_{HH} = 8.5, 7.4 Hz), 7.42 (1H, dd, J_{HH} = 8.8, 7.4 Hz), 7.11 (1H, dd, J_{HH} = 7.7, 1.0), 4.98 (2H, s), 2.87 (6H, s), 2.51 (6H, s), 2.03 (6H, s). **^{13}C NMR** (100.62 MHz, $CDCl_3$) δ (ppm): 153.1, 151.6, 136.3, 134.0, 132.7, 131.6, 131.5, 131.2, 129.7, 129.4, 129.03, 129.01, 123.5, 118.5, 115.4, 56.7, 45.7, 27.1, 13.7, 12.7. **^{19}F NMR** (376 MHz, $CDCl_3$) δ (ppm): -146.41 (td, _J_ = 62.5, 31.7 Hz)**. HR-MS (**ESI-) m/z calcd. for $C_{26}H_{26}O_2N_3BCl_2F_2S$ $[M-H]^+$ 562.1111, found 562.1106. **m. p.** = 255–260 °C.

***N,N'*-Dimethyl-5-[(2,6-dibromo-4,4-difluoro-1,3,5,7-tetramethyl-4H-3aλ4,4a-diaza-4λ4-bora-s-indacen-8-yl)methoxysulfinyl]-1-naphthylamine (4-Br)**

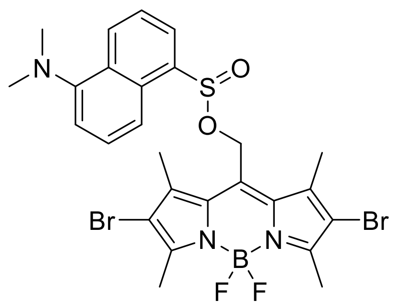

BODIPY **2-Br** (75 mg, 169 µmol, 1 eq) was dissolved in anhydrous acetone, followed by KI addition (45 mg, 26 µmol, 1.6 eq). After 10 minutes of stirring, acetone (3 mL) was removed, and the resulting solid was dissolved in a minimum amount of DMF (3 mL). Compound **3** (132 mg, 247 µmol, 1.5 eq) was dissolved in water, and the pH was adjusted to 7 by titration with NaOH to form a sodium salt. The solvent was removed, and the solid was used for the reaction with **2-Br** monitored with TLC. Full conversion was observed after 5 minutes of stirring. Then, the solvent was removed, and the crude product was purified by gradient chromatography with a DCM:CHEX mobile phase (0–100 % DCM in CHEX). The solvent was removed, yielding the target compound as orange-red crystals (24 mg, 22 %).

^{1}H NMR (400.13 MHz, $CDCl_3$) δ (ppm): 8.61 (1H, dd, J_{HH} = 8.4, 1.2 Hz), 8.30 (1H, dd, J_{HH} = 7.4, 1.3 Hz), 8.14 (1H, dd, J_{HH} = 8.6, 1.0 Hz), 7.55 (1H, dd, J_{HH} = 8.6, 7.4 Hz), 7.39 (1H, dd, J_{HH} = 8.6, 7.6 Hz), 7.09 (1H, dd, J_{HH} = 7.6, 1.0 Hz), 5.01 (2H, s), 2.86 (6H, s), 2.52 (6H, s), 2.0 (6H, s). **^{13}C NMR** (100.62 MHz, $CDCl_3$) δ (ppm): 154.5, 151.6, 138.9, 134.0, 132.7, 132.3, 131.4, 131.2, 129.5, 129.4, 129.0, 123.5, 118.4, 115.4, 113.2, 56.8, 45.8, 27.0, 15.6, 14.0. **^{19}F NMR** (376 MHz, $CDCl_3$) δ (ppm): -146.04 (m). **HR-MS** (ESI-) m/z calcd. for $C_{26}H_{26}O_2N_3BBr_2F_2S$ $[M-H]^-$ 650.0101, found 650.0094. **m. p.** = 257-260 °C.

***N,N'*-Dimethyl-5-[(2,6-diiodo-4,4-difluoro-1,3,5,7-tetramethyl-4H-3aλ4,4a-diaza-4λ4-bora-s-indacen-8-yl)methoxysulfinyl]-1-naphthylamine (4-I)**

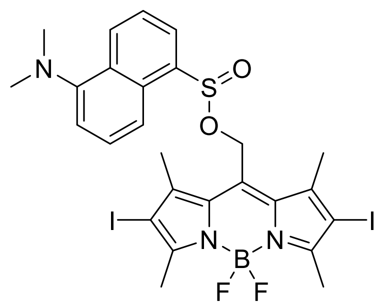

BODIPY **2-I** (65 mg, 137 µmol, 1 eq) and 5-(dimethylamino)naphthalene-1-sulfinic acid (27 mg, 137 µmol, 1 eq) were dissolved in DMF (2 mL). The reaction mixture was stirred for 10 minutes. The solvent was removed, and the mixture was dissolved in DCM and co-evaporated with SiO_2. Then, the mixture was dry-loaded on the column, and the crude product was purified by gradient chromatography using a DCM:CHEX mobile phase (0–100 % DCM in CHEX). The solvent was removed, yielding dark violet crystals (22 mg, 25 %).

^{1}H NMR (400.13 MHz, $CDCl_3$) δ (ppm): 8.60 (1H, dd, J_{HH} = 8.7, 1.0 Hz), 8.28 (1H, dd, J_{HH} = 7.4, 1.3 Hz), 8.13 (1H, dd, J_{HH} = 8.6, 1.0 Hz), 7.54 (1H, dd, J_{HH} = 8.6, 7.4 Hz), 7.38 (1H,dd, J_{HH} = 8.7, 7.7 Hz), 7.09 (1H, dd, J_{HH} = 7.7, 1.0 Hz), 5.05 (2H, s), 2.87 (6H, s), 2.55 (6H, s), 2.08 (6H, s). **^{13}C NMR** (100.62 MHz, $CDCl_3$). δ (ppm): 157.3, 151.6, 143.5, 134.0, 133.0, 132.7, 131.4, 131.2, 129.4, 129.00, 128.6, 123.5, 118.4, 115.4, 77.4, 57.2, 45.9, 19.2, 16.4. **^{19}F NMR** (376 MHz, $CDCl_3$) δ (ppm): -145.73 (m). **HR-MS** (ESI-) m/z calcd. For $C_{26}H_{26}O_2N_3BF_2I_2S$ $[M-H]^-$ 745.9823; found 745.9820. **m. p.** = 235–240 °C.

N-methyl-N-phenylmethacrylamide

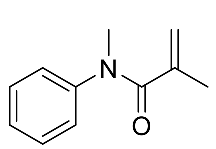

Methacryloyl chloride (1.3 mL, 9.3 mmol, 2 eq) was added to a solution of *N*-methyl aniline (0.506 mL, 4,7 mmol, 1 eq) and TEA (1.4 mL, 13.9 mmol, 3 eq) in DCM (10 mL) at 0 °C under nitrogen atmosphere. The reaction mixture precipitated, warmed to room temperature and left to stir for 6 h. The mixture was further quenched by H_2O (100 mL). Then, the crude mixture was diluted with DCM (100 mL) and washed with water (3x). The combined organic layers were dried over $MgSO_4$, and the solvents were removed. The crude product was adsorbed on the pad of celite and purified by gradient chromatography with a CHEX:EtOAc mobile phase (0–50 % of EtOAc). The solvents were removed, yielding a yellowish oil, which further crystallized (417 mg, 88 %).

^{1}H NMR (400.13 MHz, $CDCl_3$) δ (ppm) 7.35 (2H, m), 7.26 (1H, m), 7.13 (2H, m), 5.01 (2H, m), 3.35 (3H, s), 1.76 (3H, m).

^{1}H NMR spectrum corresponds to the literature.[3]

8-(hydroxymethyl)-4,4-difluoro-1,3,5,7-tetramethyl-4*H* -3aλ^4,4a-diaza- 4λ^4-bora-s-indacene (5)

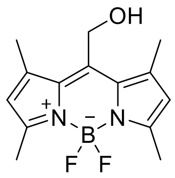

The compound was synthesized according to a previously published procedure[4]

^{1}H NMR (400.13 MHz, $CDCl_3$) δ (ppm): 6.09 (2H, s), 4.91 (2H, s), 2.53 (6H, s), 2.51 (6H, s)

The ^{1}H NMR spectrum matches spectra previously published in the literature.[4]

8-(methyl)-4,4-difluoro-1,3,5,7-tetramethyl-4*H* -3aλ^4,4a-diaza- 4λ^4-bora-s-indacene (9)

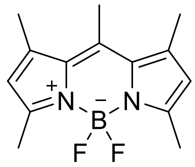

Acetyl chloride (4.4 mL, 26 mmol) was dropwise added to 2,4-dimethylpyrrole (5.4 mL, 61 mmol) in DCM (20mL) at room temperature. The resulting deep-red solution was heated to reflux for 1 h in a heating mantle. After cooling down, the mixture was poured into *n*-hexane (100 mL) and concentrated to dryness on a rotary evaporator. Without further purification, the crude intermediate was dissolved in DCM (100 mL) and TEA (10.5 mL, 75 mmol) added dropwise, and the solution was stirred at room temperature for 15 min. Next, $BF_3 \cdot Et_2O$ (13.9 mL, 112 mm) was added dropwise and stirred at room temperature for 1 h. A dark red solution was washed with a saturated Na_2CO_3 solution (4 x 100 mL). The crude product was adsorbed on the pad of celite and purified by gradient chromatography with a CHEX:DCM mobile phase (0–100 % of DCM). The orange fluorescent product was precipitated from DCM:CHX, yielding a red orange crystalline solid. (4 g, 58%).

^{1}H NMR (400.13 MHz, $CDCl_3$) δ (ppm) 6.05 (2H, s), 2.58 (3H, s), 2.52 (6H, s), 2.42 (6H, s)

^{1}H NMR spectrum matches spectra previously published in the literature.[5]

Photophysical and photochemical properties of BODIPY-sulfinates

Table S1. Photophysical and photochemical properties of BODIPY-sulfinates

	solvent	λ_{abs}	ε_{max}	λ_{fluo}	$\Delta\tilde{\nu}$	Φ_f	Φ_r
compd		(nm)	($M^{-1}cm^{-1}$)	(nm)	(cm^{-1})		
**1**[i]	DMSO/water	517	37400	534	615	0.0084 ± 0.0002	n.d.[k]
**4-H**	DCM/MeOH	524	152000	537	462	0.0073 ± 0.0006	n.r.
**4-Cl**	DCM/MeOH	552	48900	569	541	0.0065 ± 0.0004	n.r.
**4-Br**	DCM/MeOH	552	74100	576	755	0.0026 ± 0.0004	n.r.
**4-I**	DCM/MeOH	560	48200	585	763	0.00026 ± 0.00002	n.r.
**5**	DCM/MeOH	511	64900	529	665	0.68 ± 0.02	n.d.

[a] Solutions in dichloromethane/methanol (1 : 9, v/v) c ≈ 2 × 10^{-5} M, n.r. = no reaction. [b] n.d. = not determined.

Fluorescence quantum yields

All compounds were dissolved in approximately 100 µL DCM, and the concentration was adjusted with MeOH to an absorbance of 0.1—0.2 at 410 nm. Then, the emission spectrum was measured (λ_{exc} = 410 nm, slits = 5 nm, spectra accumulation = 1, integration time = 1 for compound **1** or 10 s for sulfinates). The peak area was calculated from the measured spectrum. The measurement was repeated 4 times with independent samples. The same process was performed for the standard (**1-OAc**) with a known fluorescence quantum yield ϕ_{std}, determining the slope of the curve of the variation of peak area as a function of absorbance at 410 nm. The quantum yield ϕ_x was calculated using the following equation.

$$\phi_x = \phi_{std}\left(\frac{\text{slope}_x}{\text{slope}_{std}}\right)$$

Due to the presence of a highly emissive impurity (**9**), the quantum yields were corrected using the standard addition method. The content of compound **1** was calculated based on the NMR purity. Then, compound **9** was added as a standard (0.25, 0.5, 0.75, 1.0 eq). Thanks to the known amount of impurity, the corrected peak area of the sample was calculated. Subsequently, the ratio ($\text{area}_{\text{REAL}}/\text{area}_{\text{CORR}}$) = 1.5 was calculated, and the quantum yields were corrected using this factor.

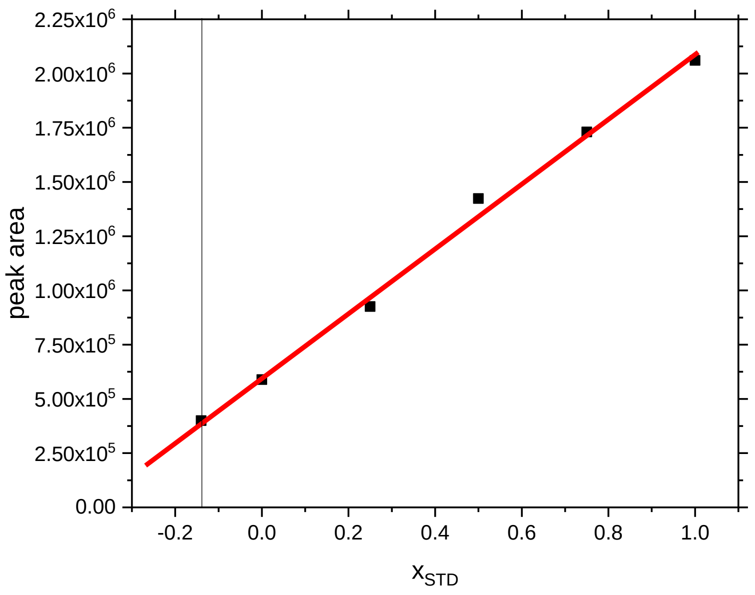

Figure S1. Standard addition method for quantum yield correction

Photoreaction quantum yields

A solution of **1** in DCM/MeOH mixture (1:9, v/v, $c \approx 2 \times 10^{-5}$ M) was irradiated at 525 nm for a defined time period. Then, HPLC spectra were measured by collecting 2 µL of the irradiated solution, adding an internal standard (5,5-difluoro-10-(hydroxymethyl)-1,3,7,9-tetramethyl-5*H*-4λ^4,5λ^4-dipyrrolo[1,2-c:2',1'-*f*][1,3,2]diazaborinine-2,8-disulfonic acid), and diluting to 20 µL in MeOH. The quantum yield was determined using an indolyl fulgide as an actinometer, according to a previously published protocol.[6]

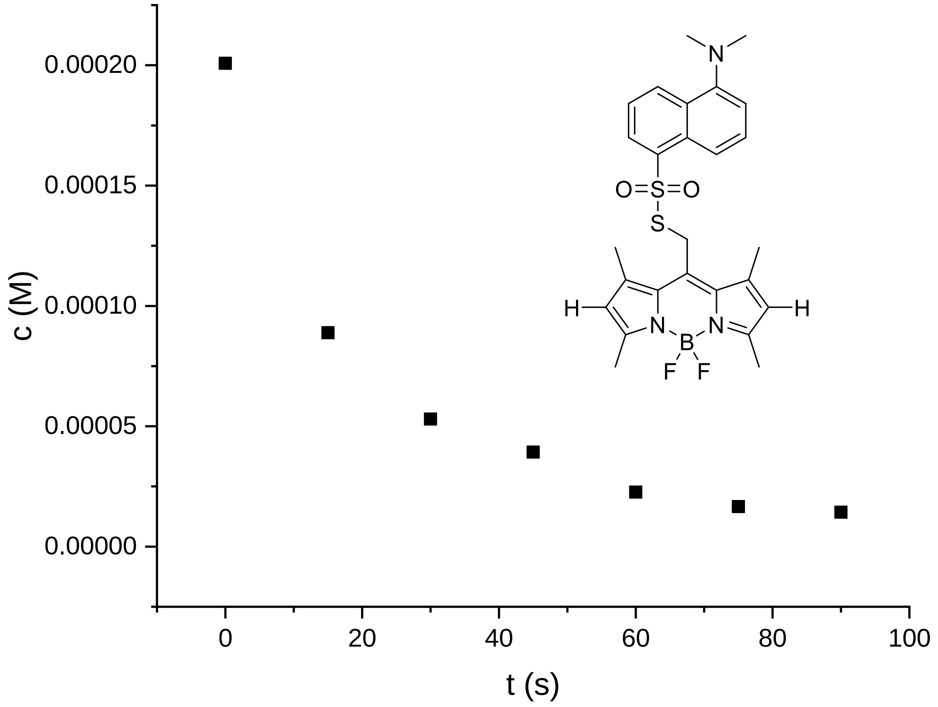

Figure S2. Variation of the concentration (**1**) as a function of time upon irradiation at 525 nm in aerated methanol (MeOH/DCM, 1/1, v/v)). Starting concentration: $c \approx 2 \times 10^{-5}$ M. The concentration was determined by UV-vis absorption and by HPLC with a standard.

Thioxanthone (TX) sensitization

Compound **1** ($c \approx 3$ µM) was irradiated at 400 nm in the presence of TX in a mixture of MeCN:MeOH:DMSO (1:2:0.001, v/v). The solution was degassed prior to irradiation using the freeze-pump-thaw method.

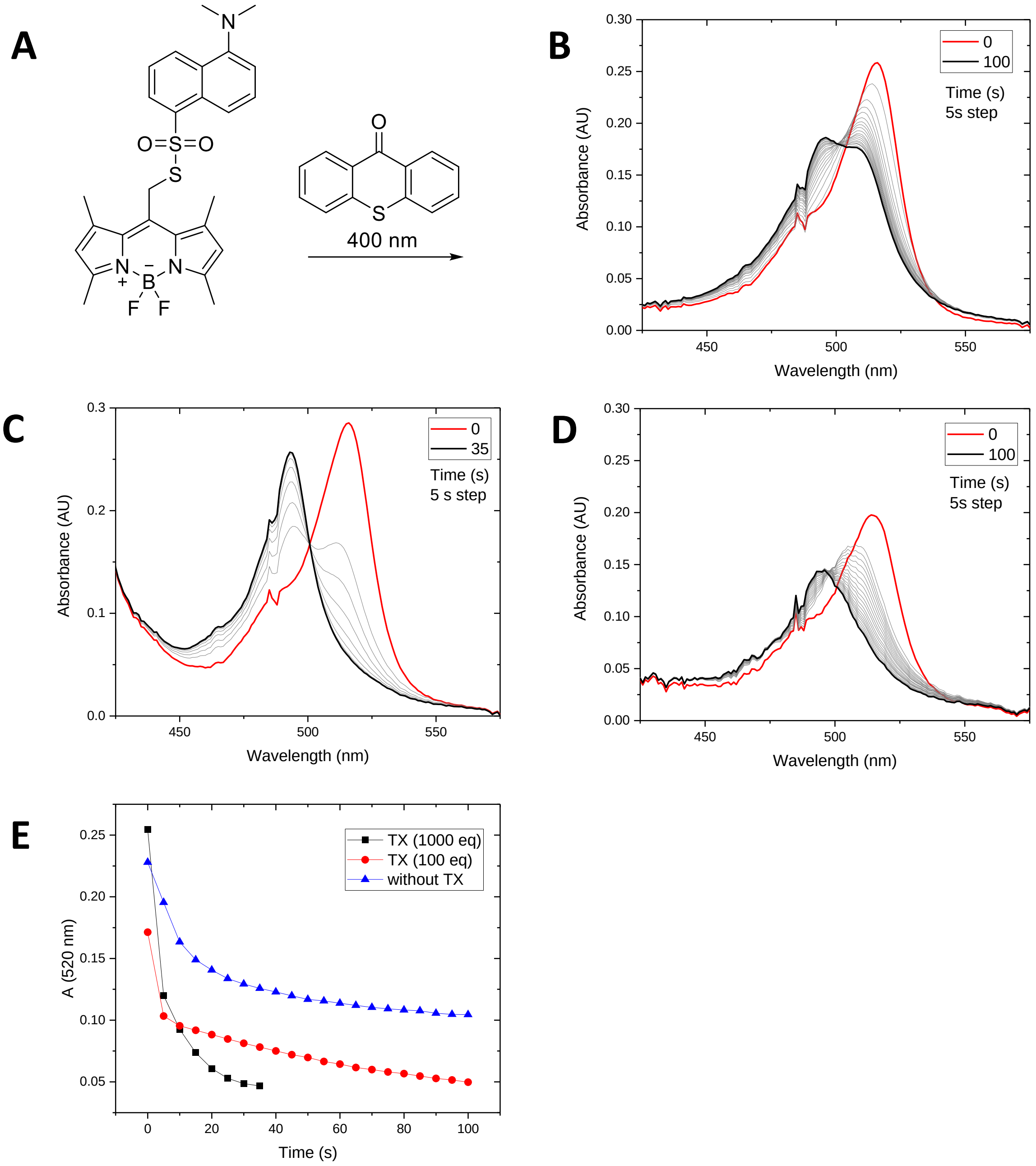

Figure S3. a) Reaction scheme. UV-Vis spectra of **1** irradiated at 400 nm in degassed MeOH: b) without TX; c) with TX (1000 eq); d) with TX (100 eq). e) Time-dependent absorption of **1** at 520 nm with and without TX.

Cyclooctatetraene (COT) quenching

Compound **1** ($c \approx 3$ µM) was irradiated at 525 nm with COT (2600 eq) in a mixture of MeOH:DMSO (1:0.003, v/v). The solution was degassed prior to irradiation using the freeze-pump-thaw method.

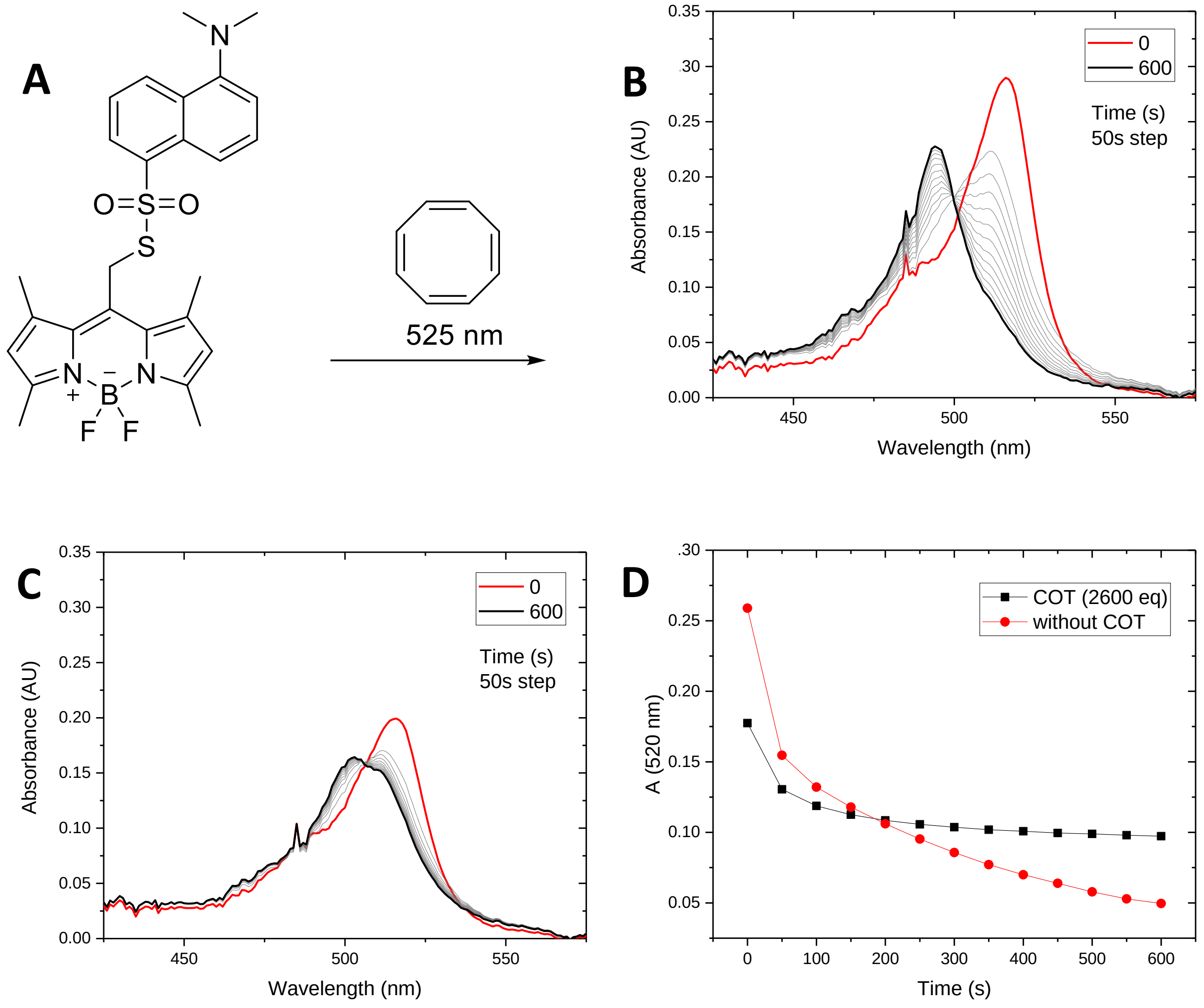

Figure S4. a) Reaction scheme of triplet quenching. UV-Vis spectra of **1** irradiated at 525 nm in degassed MeOH: b) without COT; c) with COT (2600 eq). d) Time-dependent absorption of **1** at 520 nm with and without COT.

H_2S yield determination

The procedures for methylene blue (MB) reagent mixture preparations, H_2S calibration curves, and NaSH standardization were adapted from protocols previously published in the literature.[7] A solution of **1** (0.5 mL, 300 μM) in a MeOH:DMSO mixture (1:0.03, v/v) was placed in an aluminum-capped vial, bubbled with argon for 10 min, and irradiated at 525 nm for 30 min, subsequently adding a solution of glutathione (corresponding concentration, 0.5 mL in PBS). The variation of the yield as a function of the incubation time is shown in Figure S4c. UV-Vis spectra were recorded after 1 h of reaction upon adding a methylene blue cocktail solution (1 mL),. Absorbance at 670 nm was used to calculate the concentration according to H_2S calibration curves (Figure S4a). Methylene blue cocktail was freshly prepared before every use by mixing zinc acetate (1 % w/v), $FeCl_3$ (30 mM in 1.2 M HCl_{aq}), and *N,N*-dimethyl-*p*-phenylene diamine (20 mM in 7.2 M HCl_{aq}) in a 1:2:2 ratio.

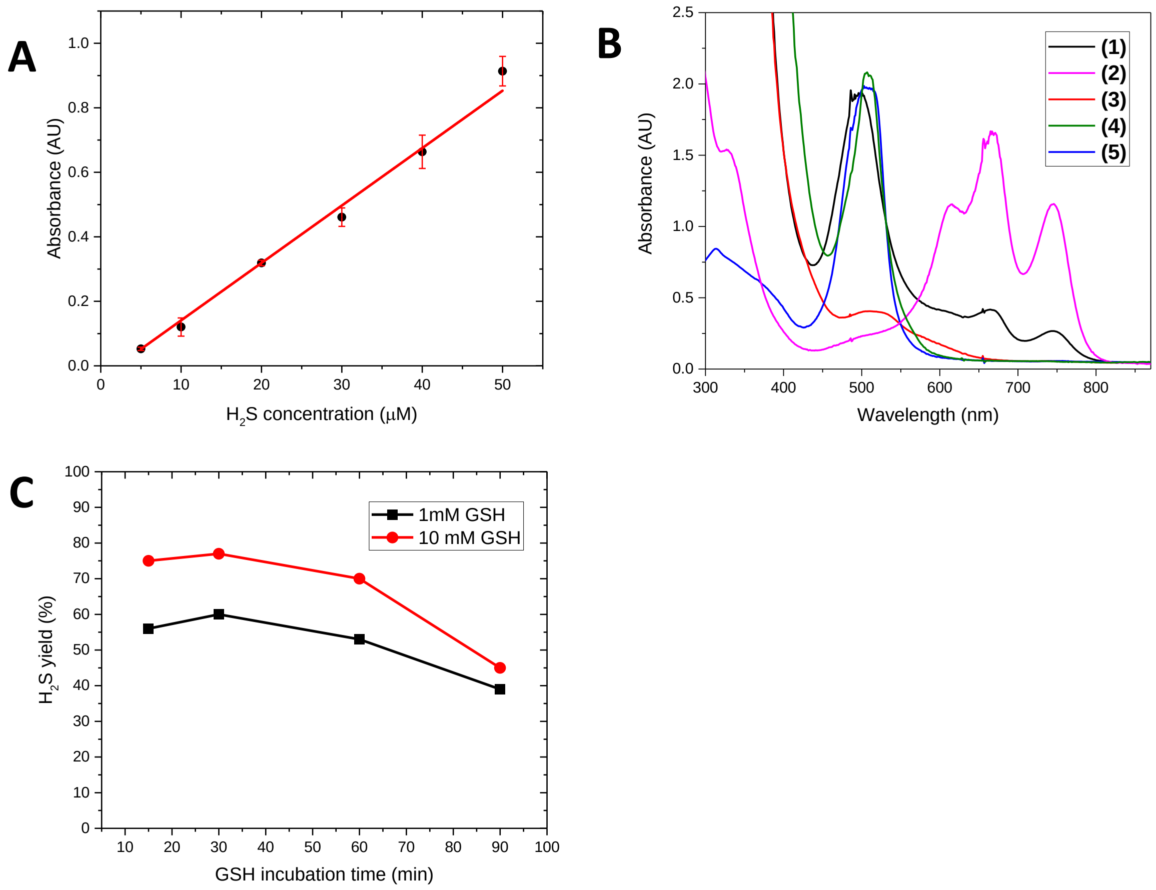

Figure S5. a) H_2S calibration curve: variation of absorption (670 nm) with the concentration of an H_2S standard; b) UV-Vis spectra of **(1)** **1** (c = 0.3 mM) irradiated at 525 nm, incubated with GSH (c = 1 mM) and MB cocktail, **(2)** NaSH (c = 0.1 mM) incubated with MB cocktail, **(3)** GSH incubated with MB cocktail, **(4)** **1** (c = 0.3 mM) irradiated at 525 nm and incubated with MB cocktail, **(5)** **1**

irradiated at 525 nm and incubated with GSH; c) Time-dependent yields of H_2S upon incubation of **1** with different concentrations of GSH

Sulfonothioate sensitization

A solution of compounds **3** (0.3 mM) and **7** (3 mM) in 0.5 mL MeOH (with 3 % water) was irradiated at 525 nm for 60 min in aluminum-capped vials bubbled with argon prior to irradiation. The resulting mixture was analyzed by HPLC-MS. The conversion of **3** to **8** was 20% in 60 min.

Scheme S1. Sensitization experiment

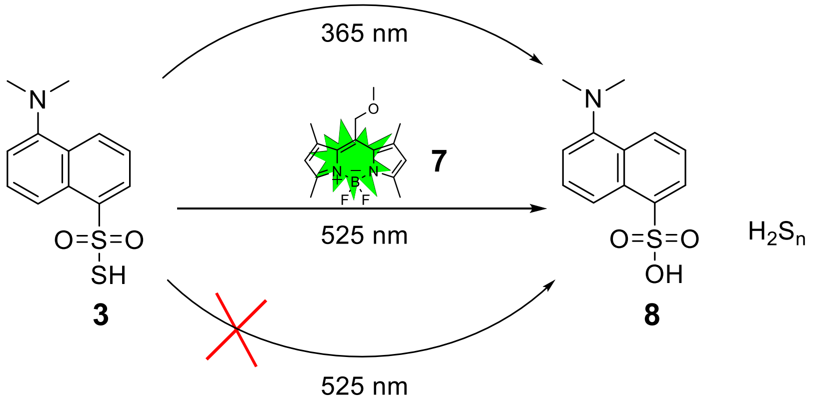

Labeling by $D_2{}^{18}O$

Compound **1** in a CD_2Cl_2:MeOD:$H_2{}^{18}O$ (5:3:2, v/v) aerated mixture was irradiated with a 525 nm LED. The ^{18}O-labeled compounds were not detected by HRMS.

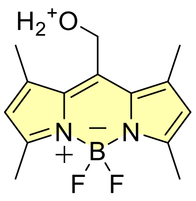

Chemical Formula: $C_{14}H_{18}BF_2N_2O^+$
Exact Mass: 279.1475

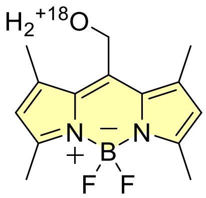

Chemical Formula: $C_{14}H_{18}BF_2N_2{}^{18}O^+$
Exact Mass: 281.1517

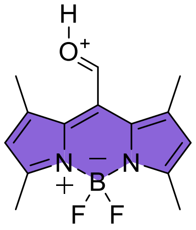

Chemical Formula: $C_{14}H_{16}BF_2N_2O^+$
Exact Mass: 277.1318

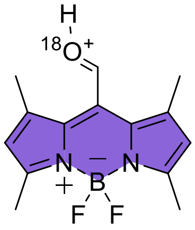

Chemical Formula: $C_{14}H_{16}BF_2N_2{}^{18}O^+$
Exact Mass: 279.1361

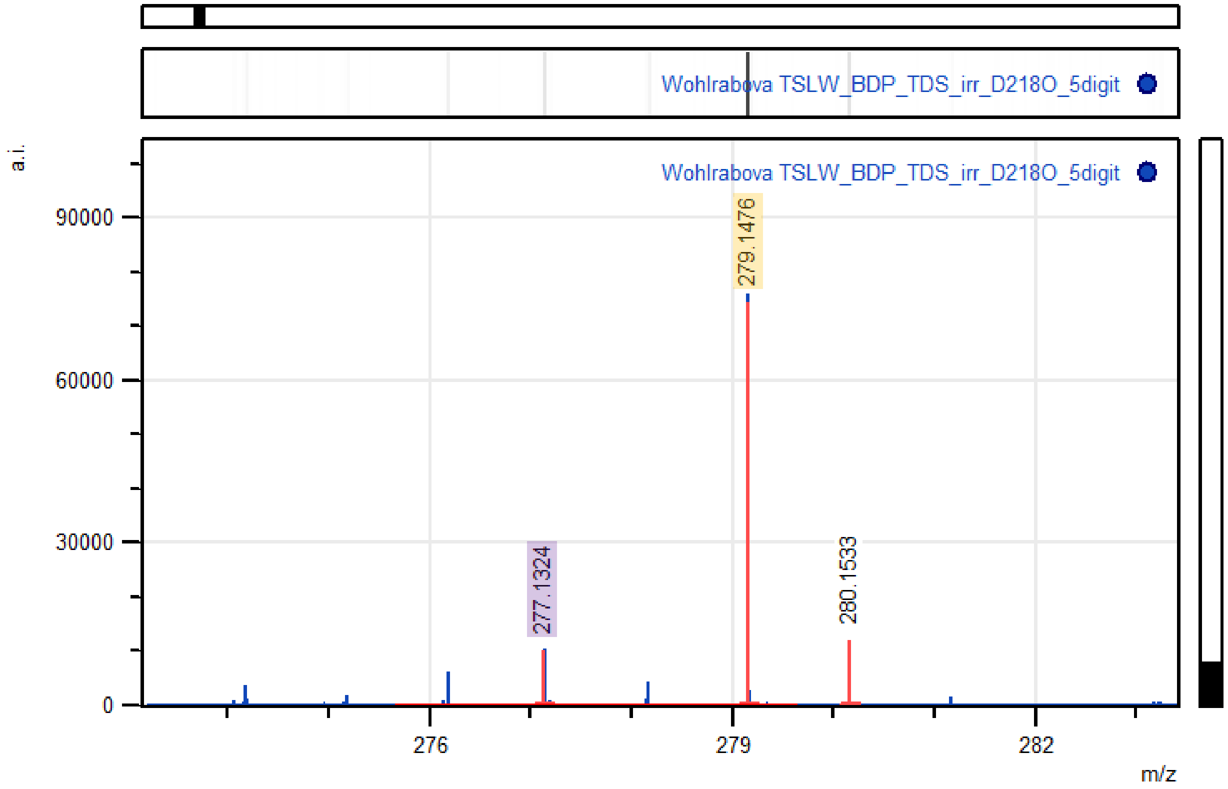

Figure S6. A zoomed HR-MS spectrum of the crude mixture obtained after irradiating **1** in $D_2{}^{18}O$ (273-283 m/z) at 525 nm. The number highlighted in violet corresponds to the non-isotopically labeled aldehyde, and the assigned value in yellow corresponds to non-isotopically labeled alcohol.

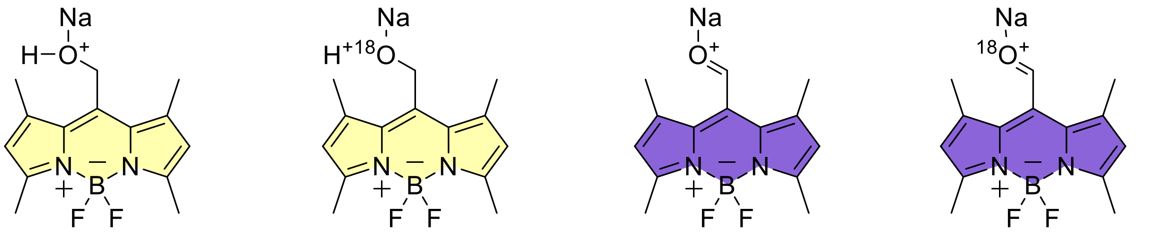

Chemical Formula: $C_{14}H_{17}BF_2N_2NaO^+$
Exact Mass: 301.1294

Chemical Formula: $C_{14}H_{17}BF_2N_2Na^{18}O^+$
Exact Mass: 303.1337

Chemical Formula: $C_{14}H_{15}BF_2N_2NaO^+$
Exact Mass: 299.1138

Chemical Formula: $C_{14}H_{15}BF_2N_2Na^{18}O^+$
Exact Mass: 301.1180

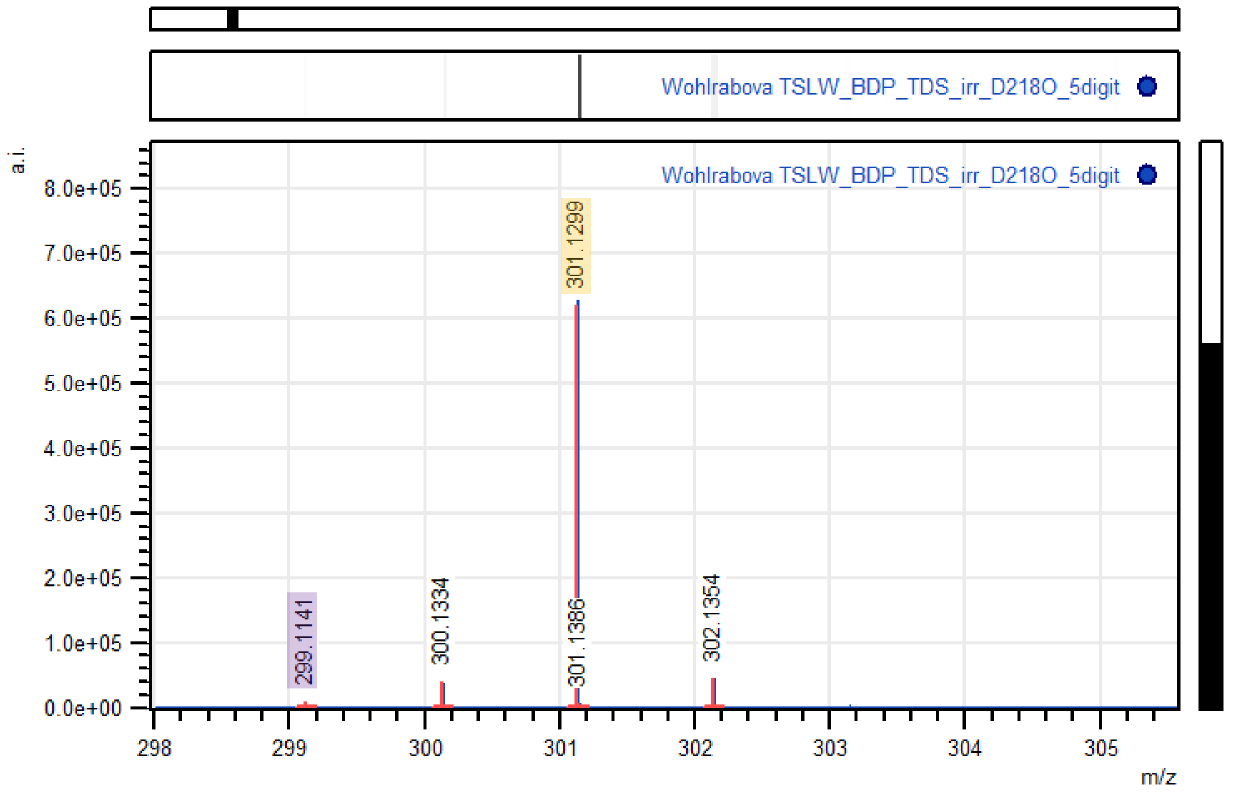

Figure S7. A zoomed HR-MS spectrum of the crude mixture after irradiating **1** with 525 nm light in $D_2{}^{18}O$ (298-306 m/z). The number highlighted in violet corresponds to the non-isotopically labeled aldehyde, and the assigned value in yellow corresponds to non-isotopically labeled alcohol.

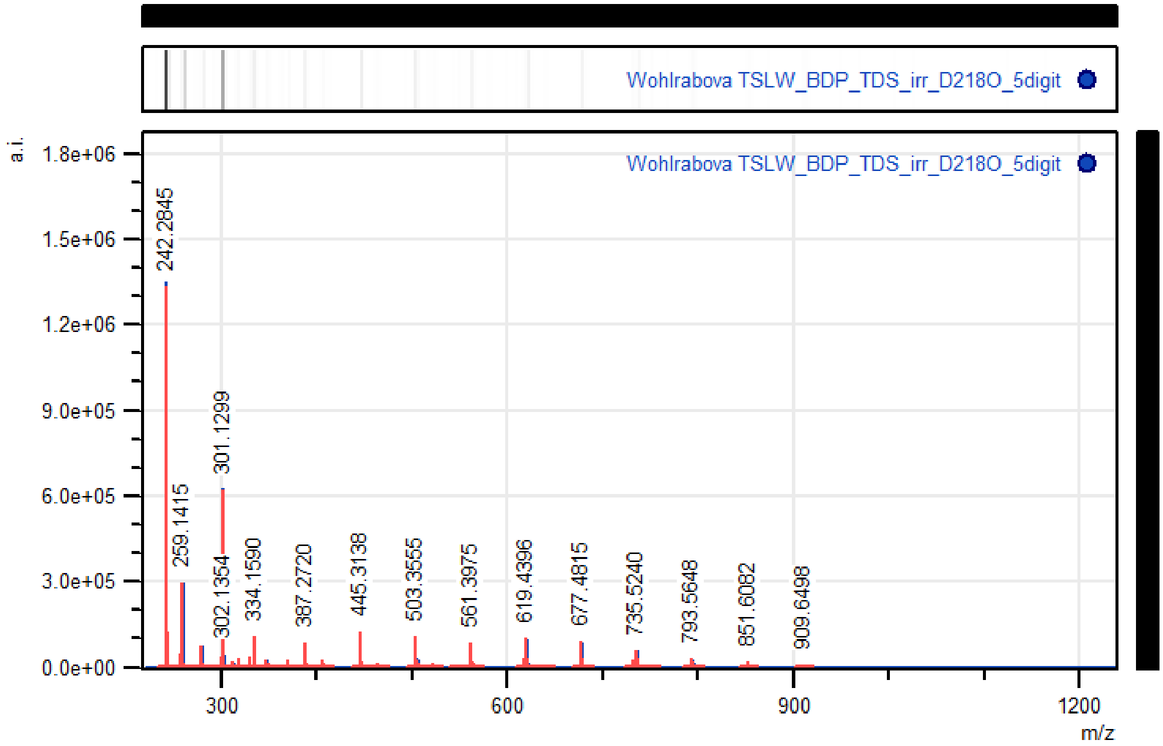

Figure S8. HR-MS spectrum of the crude mixture after irradiating **1** with 525 nm light in $D_2{}^{18}O$

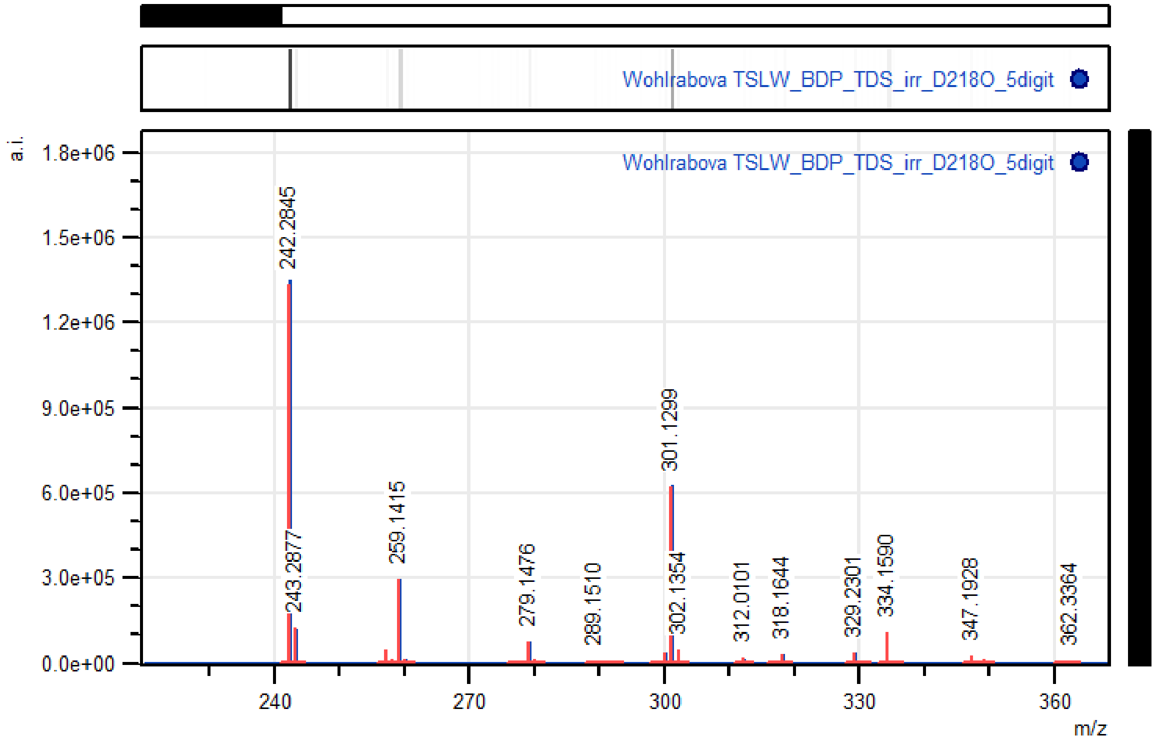

Figure S9. Zoomed HR-MS spectrum of the crude mixture after irradiating **1** with 525 nm light in $D_2{}^{18}O$

Spin trapping

N-Methyl-*N*-phenylmethacrylamide (c = 0.95 mM) was added to a solution of **1** (c = 0.95 mM) in DCM:MeOH mixture (1:9). Then, the mixture was irradiated with 525 nm LED for 3 min, followed by HPLC-MS analysis. The experiment was performed under aerated conditions.

Scheme S2. Spin-trapping of radicals using *N*-methyl-*N*-phenyl-methacrylamide[8]

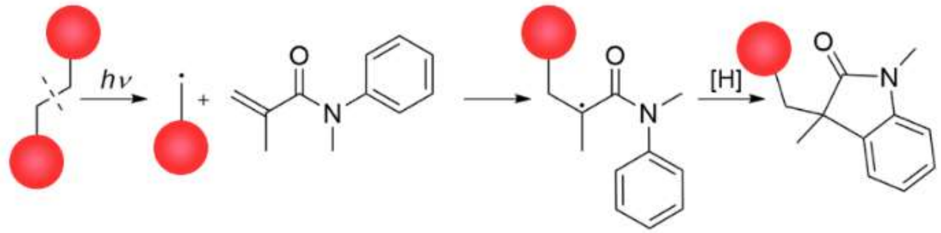

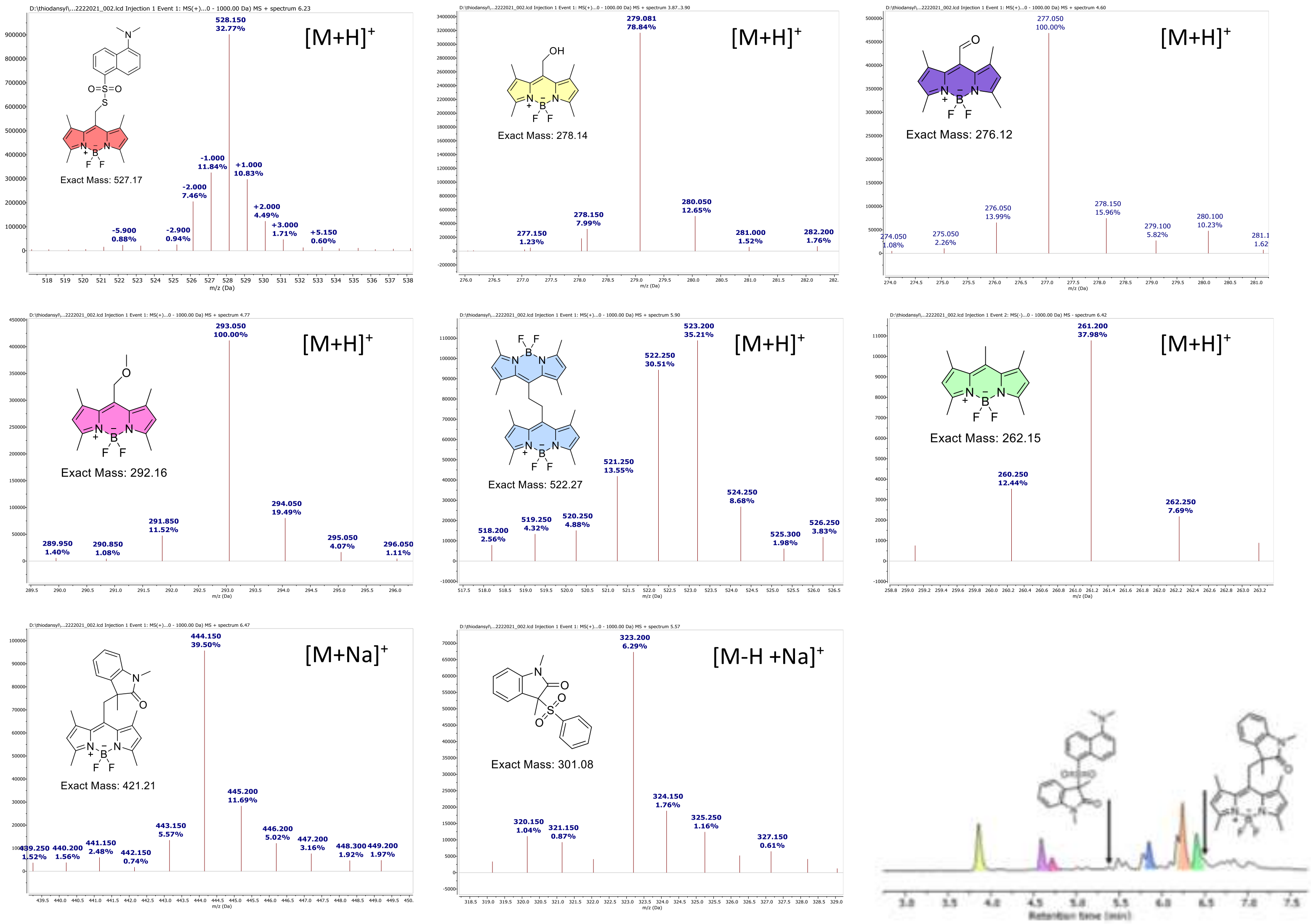

Figure S10. LR-MS spectra of photoproducts from **1** irradiation in the presence of *N*-methyl-*N*-phenylmethacrylamide

Photoreactivity and hydrolytic stability of 3

The photostability of **3** was analyzed by irradiating the compound with a 365 nm light source in a H_2O:MeOH mixture (1:1, v/v). The reaction mixture was further analyzed by HPLC-MS. The hydrolytic stability of **3** was assessed at 50 °C in H_2O:MeOH mixture (1:1, v/v), and the solution was further analyzed by HPLC-MS. The compound is stable for more than 24 h in solution at room temperature in the dark.

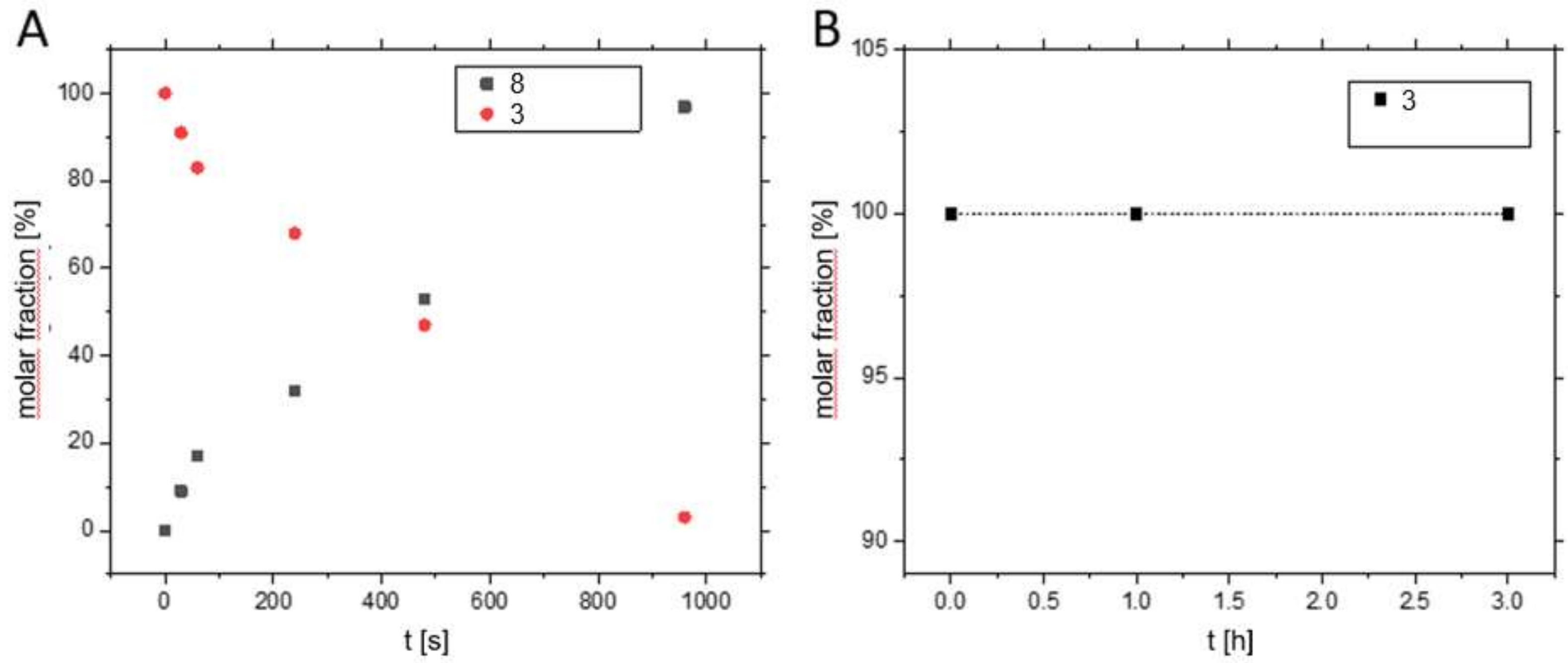

Figure S11. A) Photoreactivity of **3**. **B)** Hydrolytic stability of **3** at 50 °C

Determination of the H_2S_2 yield

The procedures for preparing the fluorescent probe **DSP-3**, constructing H_2S_2 calibration curves, and determining the H_2S_2 yield were adapted from protocols previously published in the literature.[9] A solution of compounds **3** (0.5 mM) and **DSP-3** (1 mM) in 1 mL MeCN:PBS (3:2, v/v, with 6 % DMSO) was irradiated at 365 nm for 60 min in aluminum-capped vials bubbled with argon prior to the irradiation. Fluorescence spectra were recorded, and the H_2S_2 yield was calculated using the calibration curves.

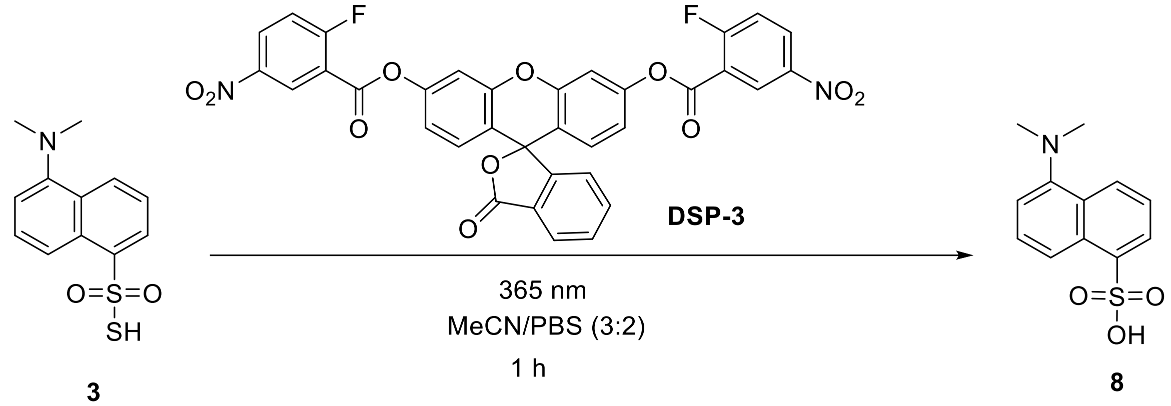

Scheme S3 Determination of the H_2S_2 yield

^{1}H NMR, ^{13}C NMR, and HR-MS spectra

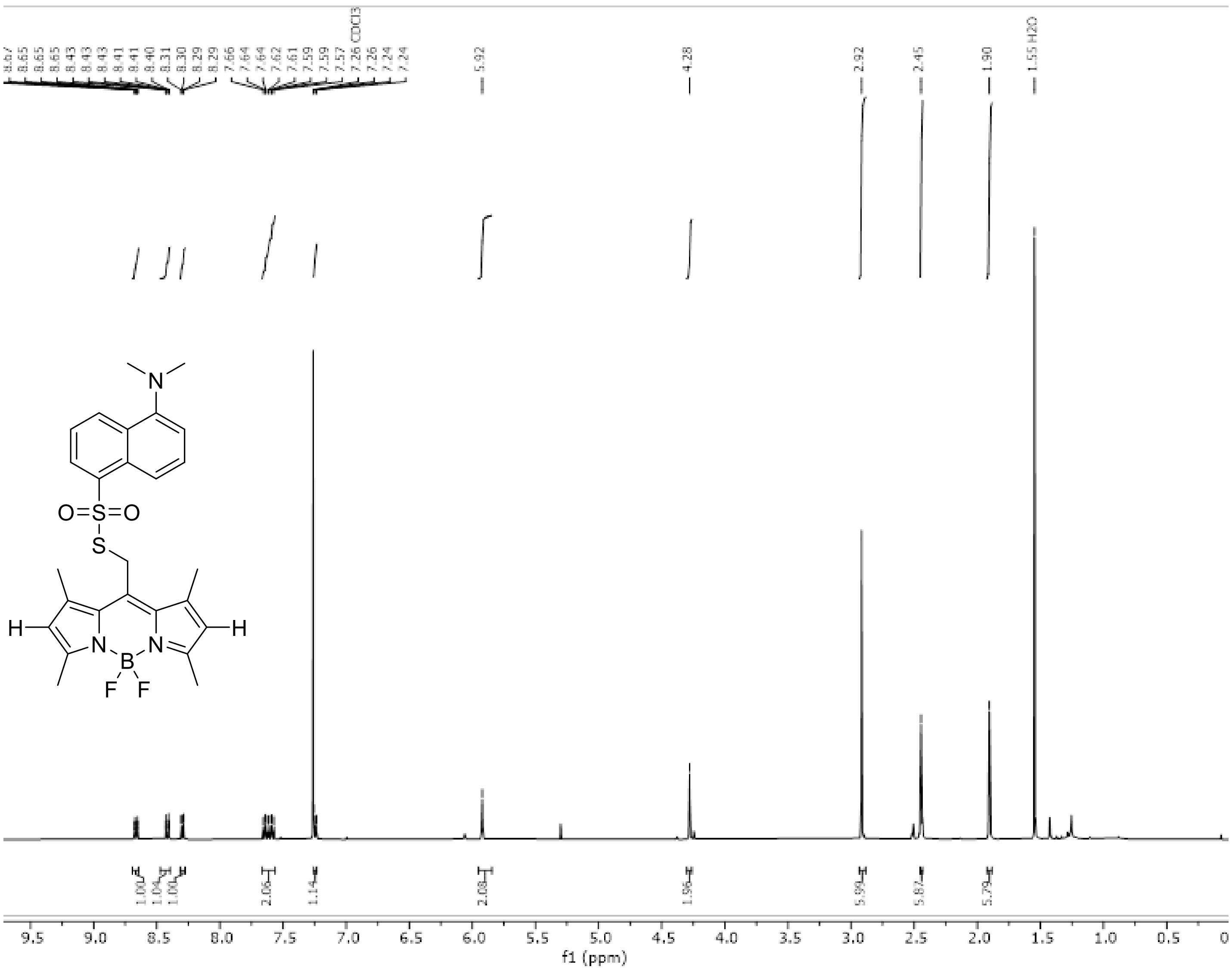

Figure S12. ^{1}H NMR (400.13 Hz) of **1** in $CDCl_3$

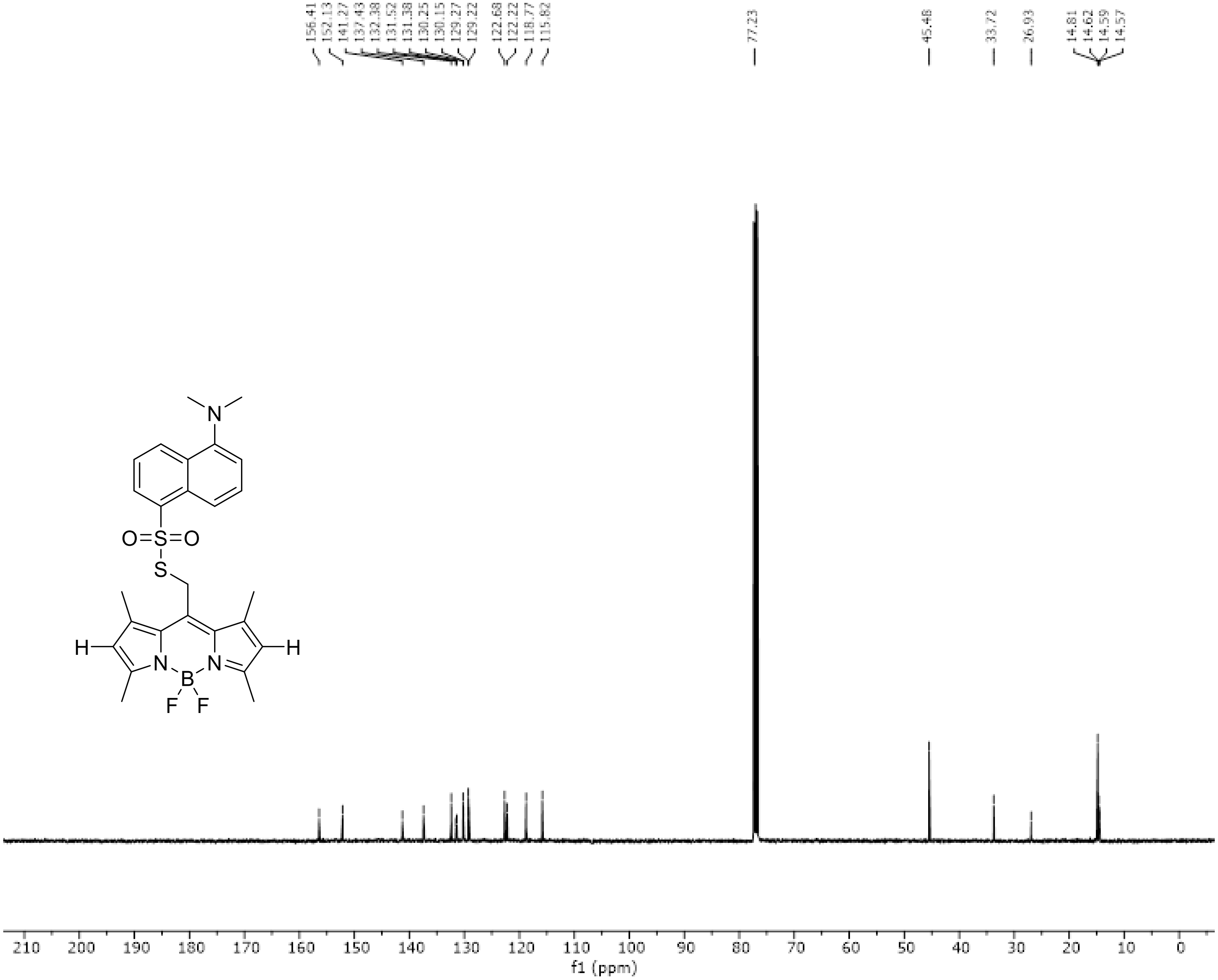

Figure S13. ^{13}C NMR (100.62 Hz) of **1** in $CDCl_3$

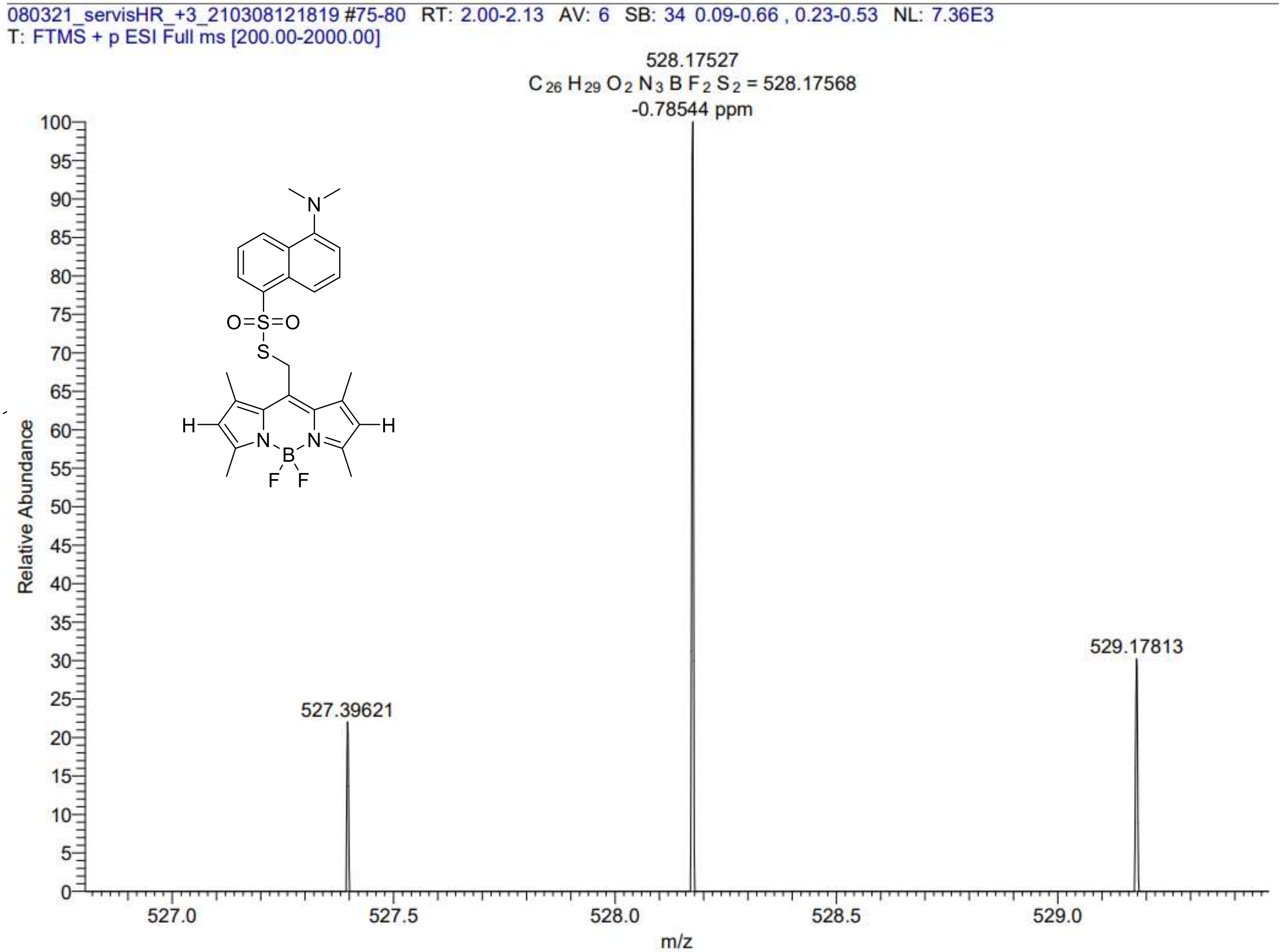

Figure S14. HRMS (ESI+) spectrum of **1**

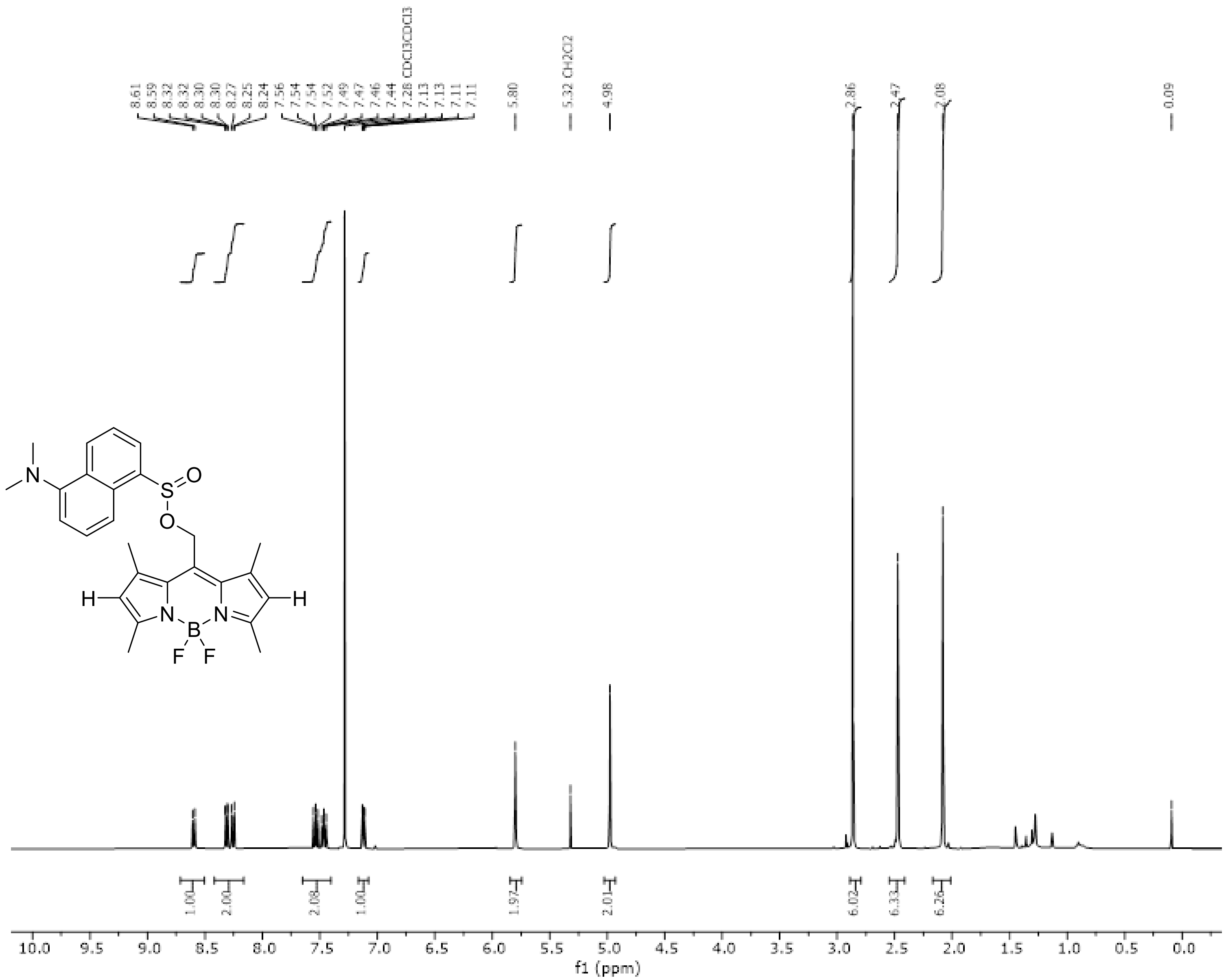

Figure S15. ^{1}H NMR (400.13 Hz) of **4-H** in $CDCl_3$

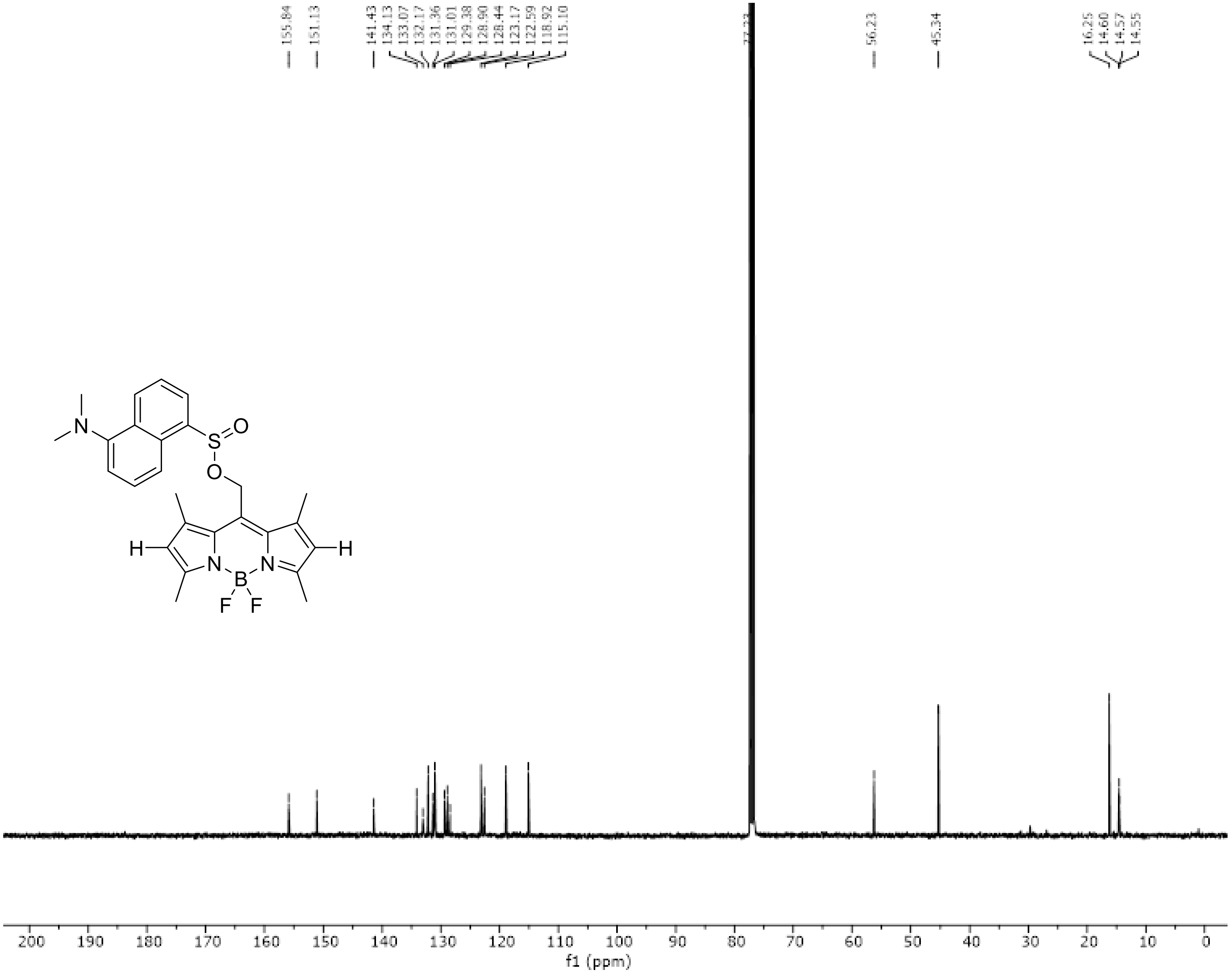

Figure S16. ^{13}C NMR (100.62 Hz) of **4-H** in $CDCl_3$

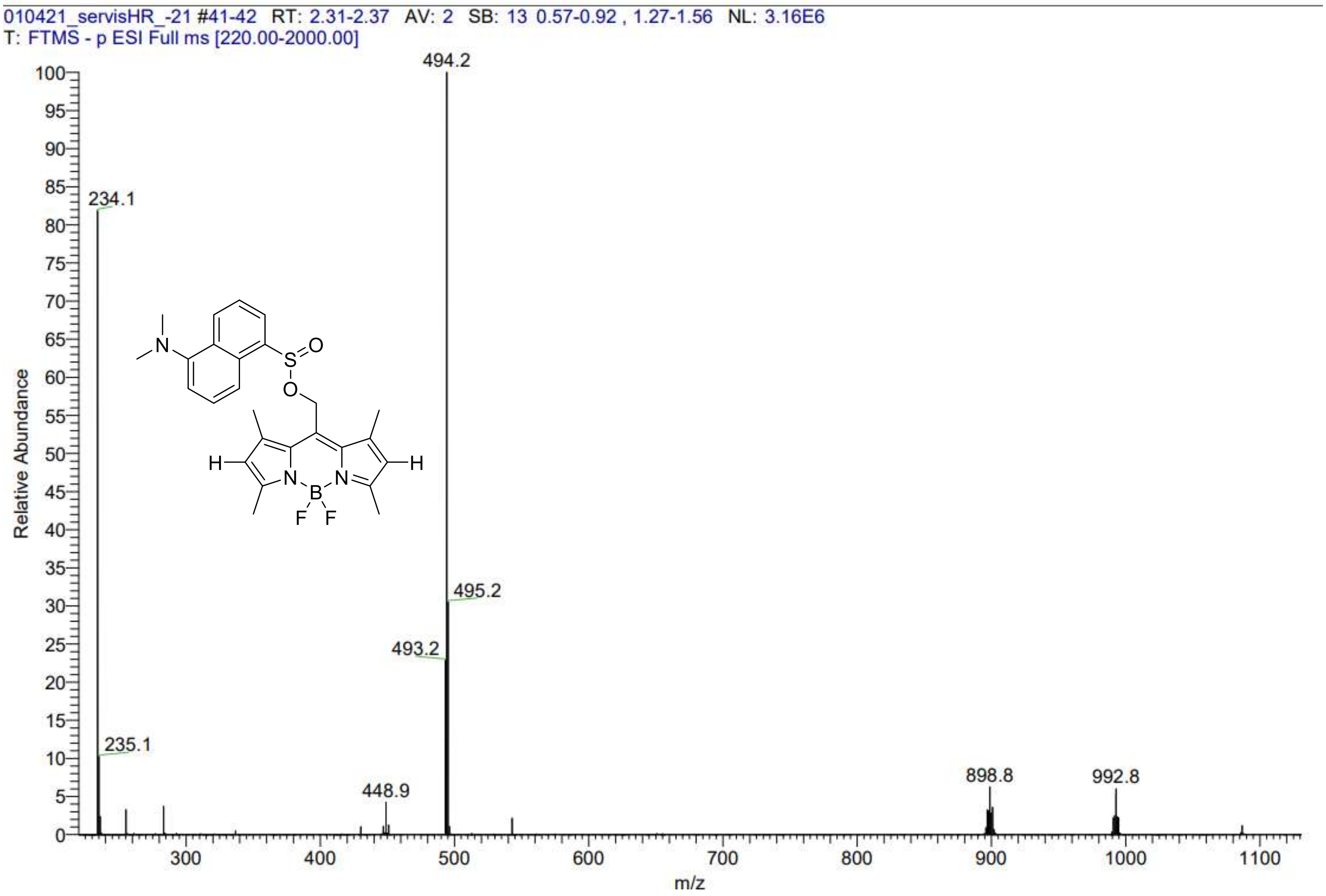

Figure S17. HRMS (ESI-) spectrum of **4-H**

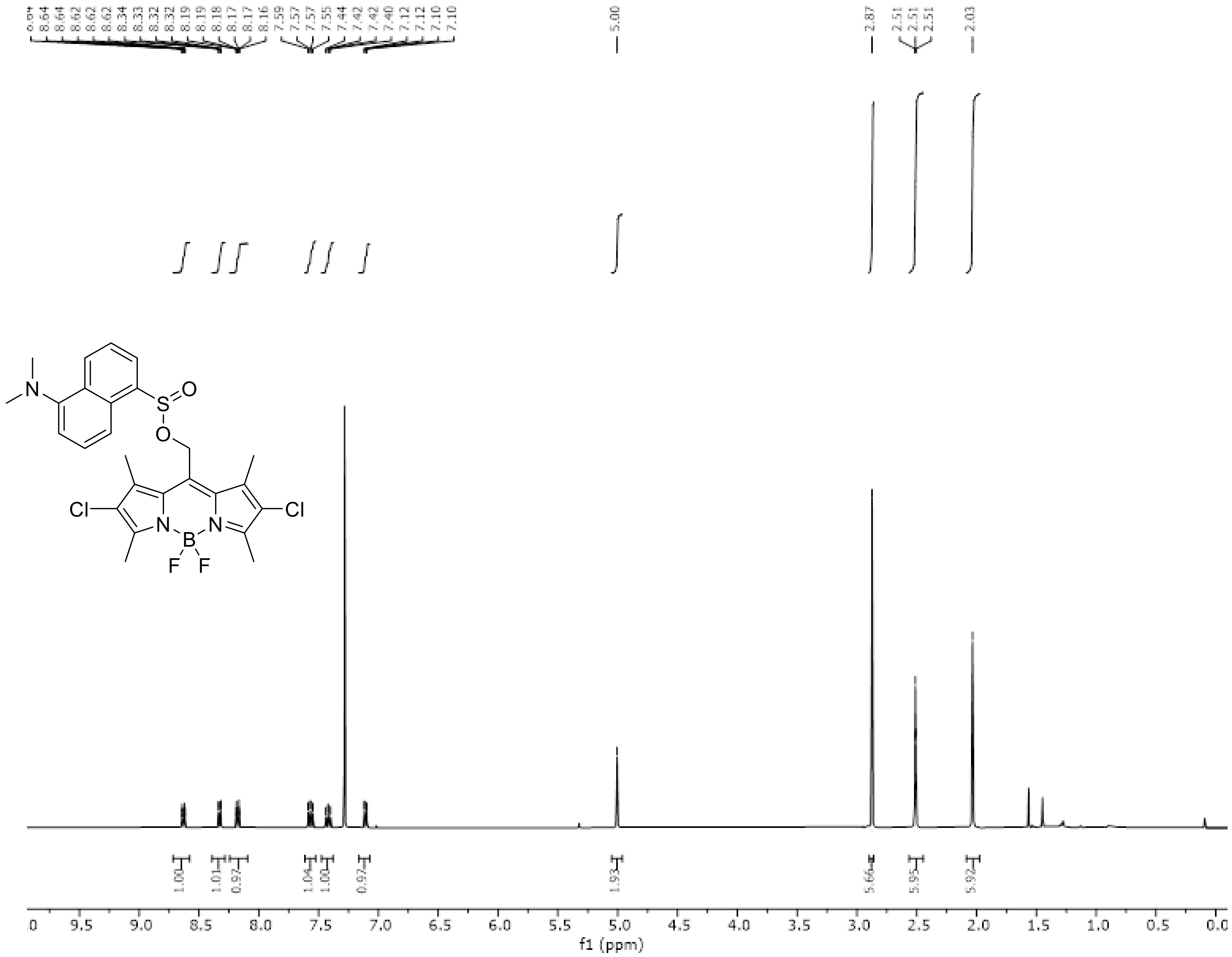

Figure S18. ^{1}H NMR (400.13 Hz) of **4-Cl** in $CDCl_3$

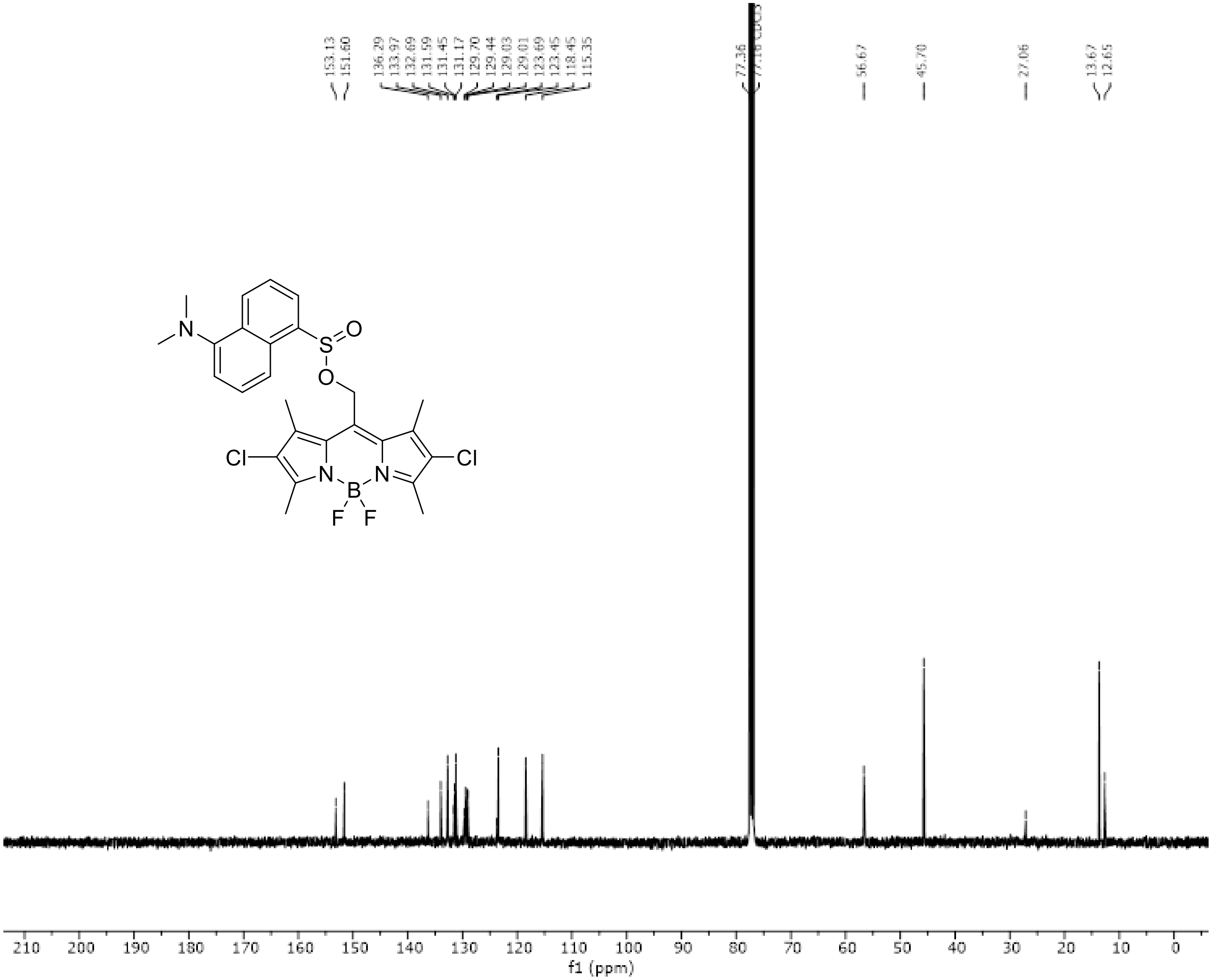

Figure S19. ^{13}C NMR (100.62 Hz) of **4-Cl** in $CDCl_3$

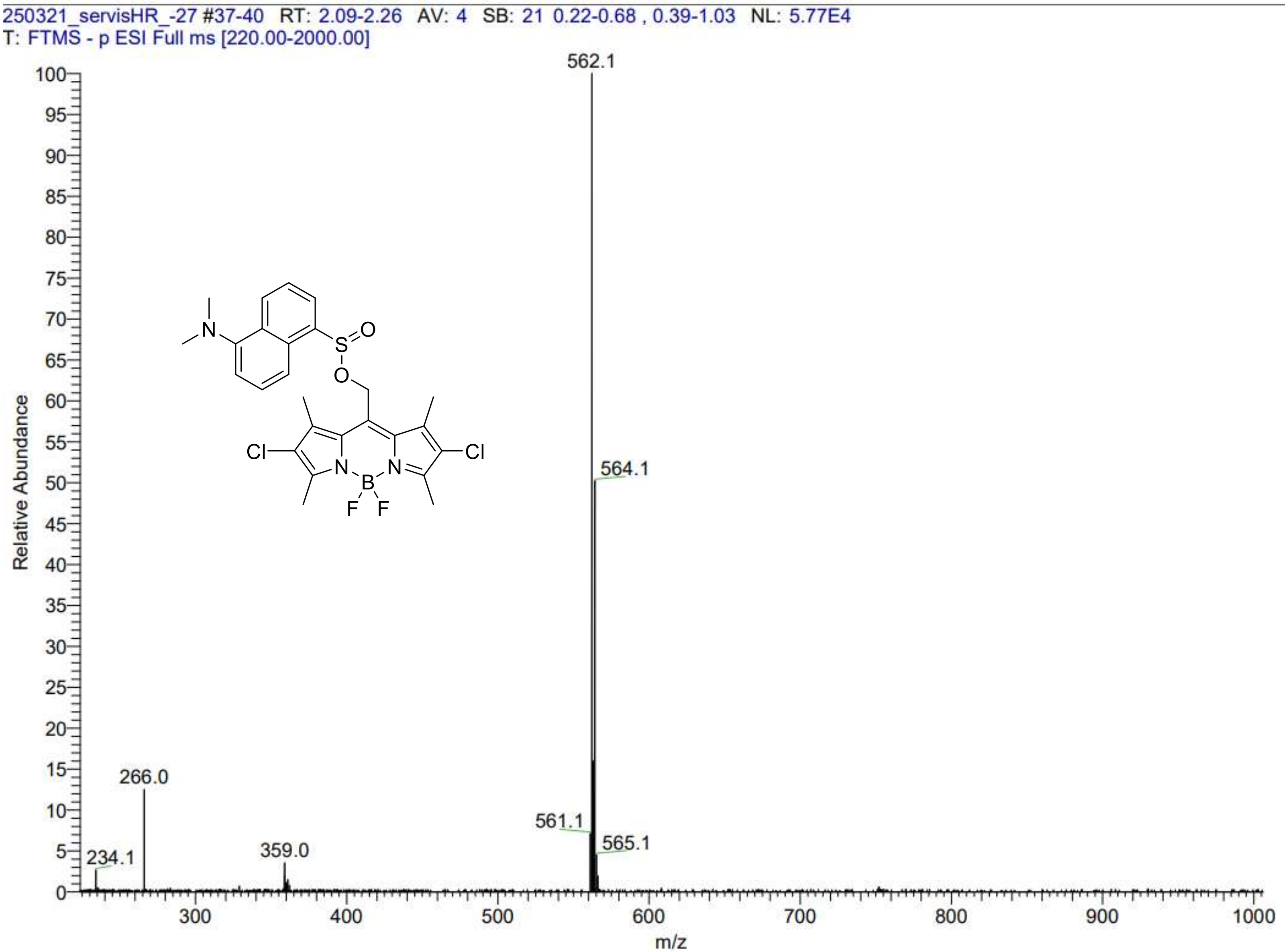

Figure S20. HRMS (ESI-) spectrum of **4-Cl**

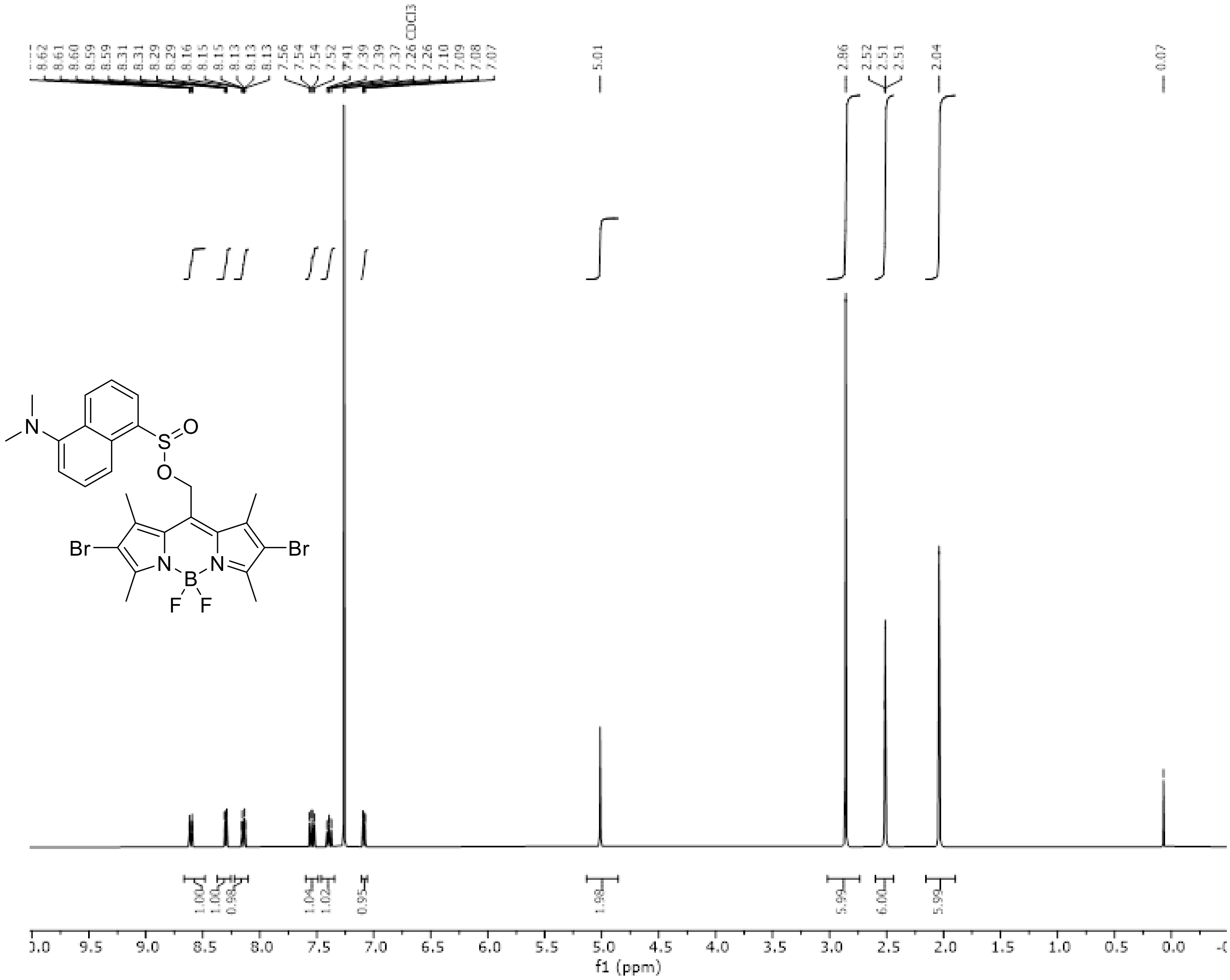

Figure S21. ^{1}H NMR (400.13 Hz) **4-Br** in $CDCl_3$

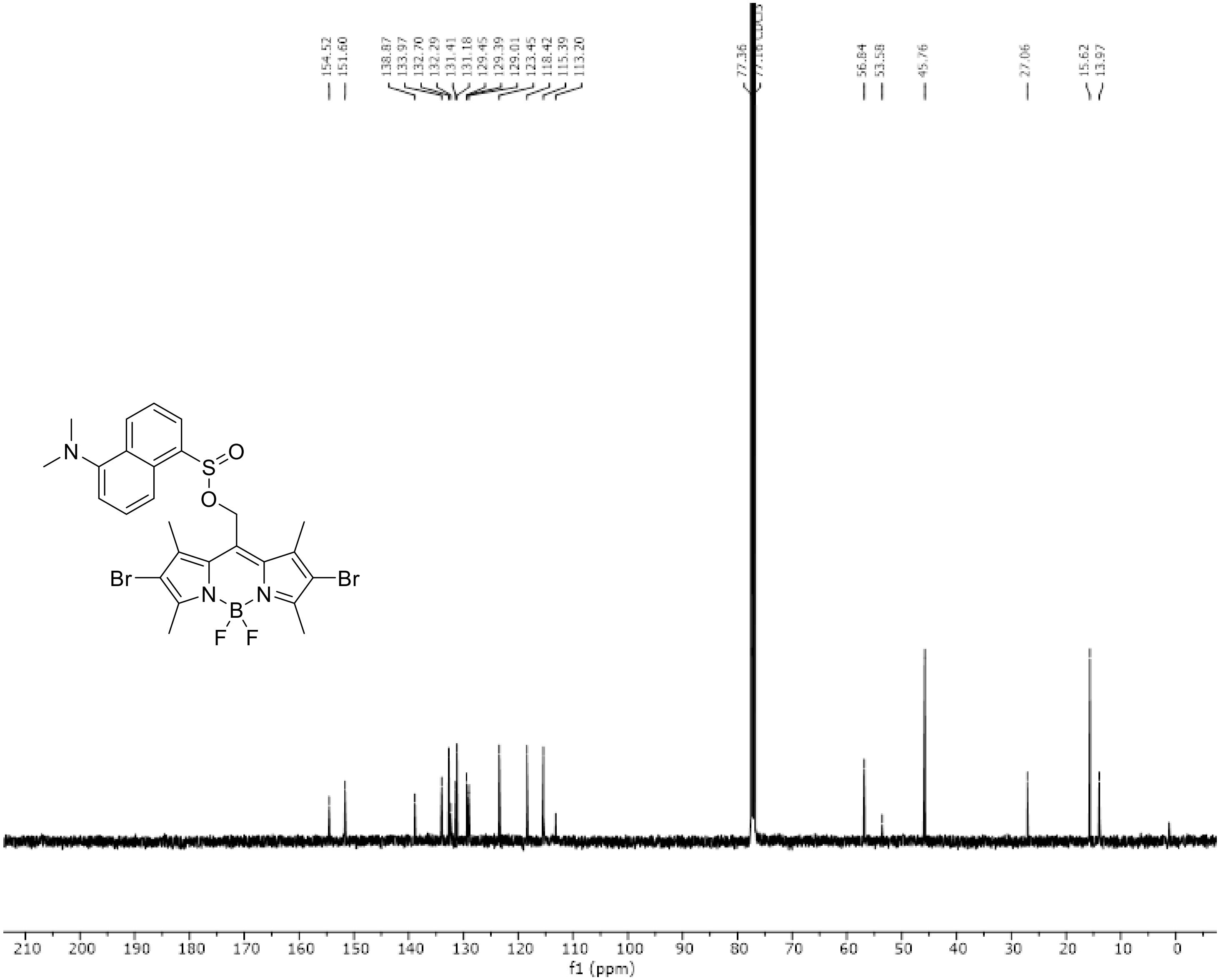

Figure S22. ^{13}C NMR (100.62 Hz) of **4-Br** in $CDCl_3$

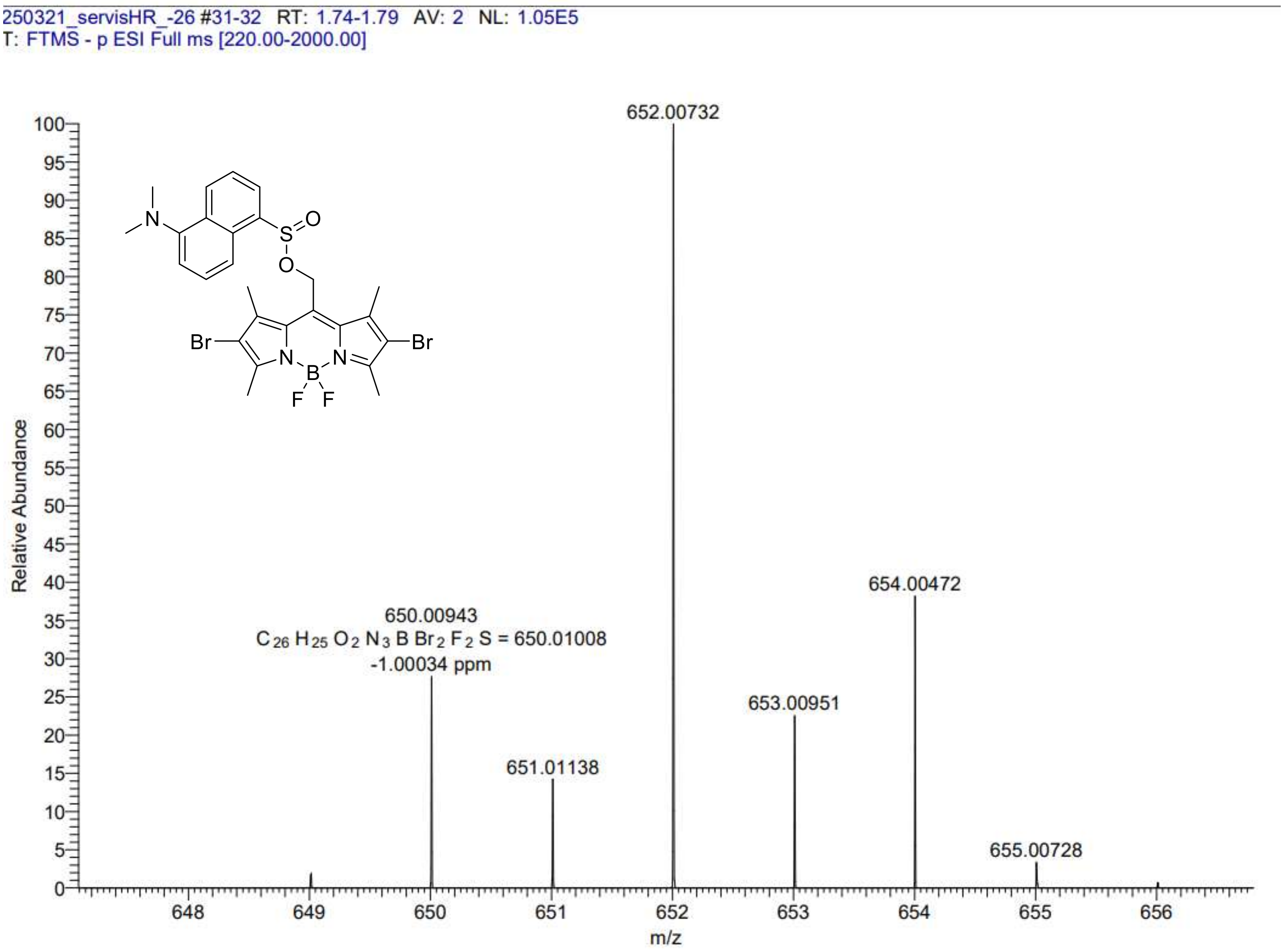

Figure S23. HRMS (ESI-) spectrum of **4-Br**

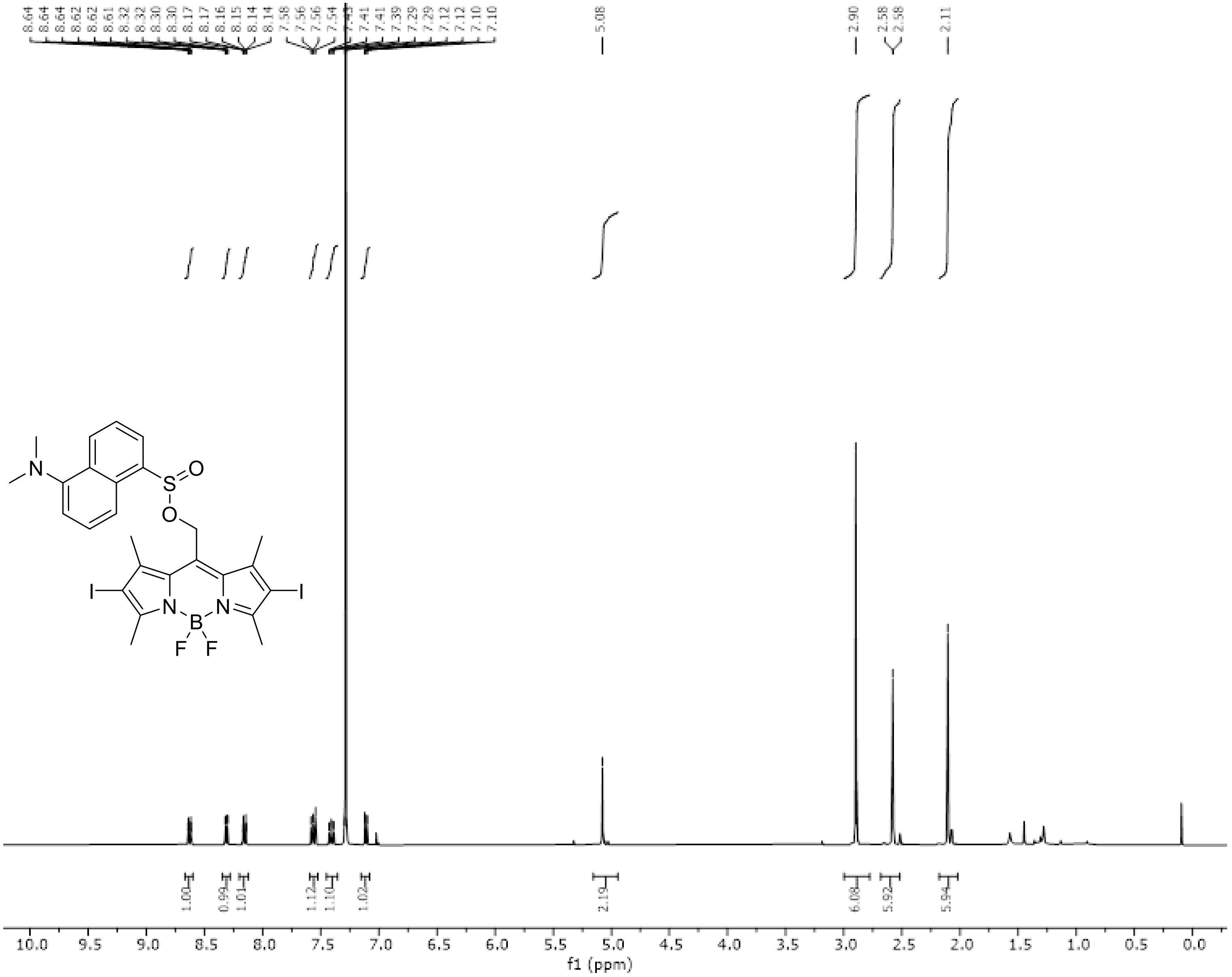

Figure S24 ^{1}H NMR (400.13 Hz) of **4-I** in $CDCl_3$

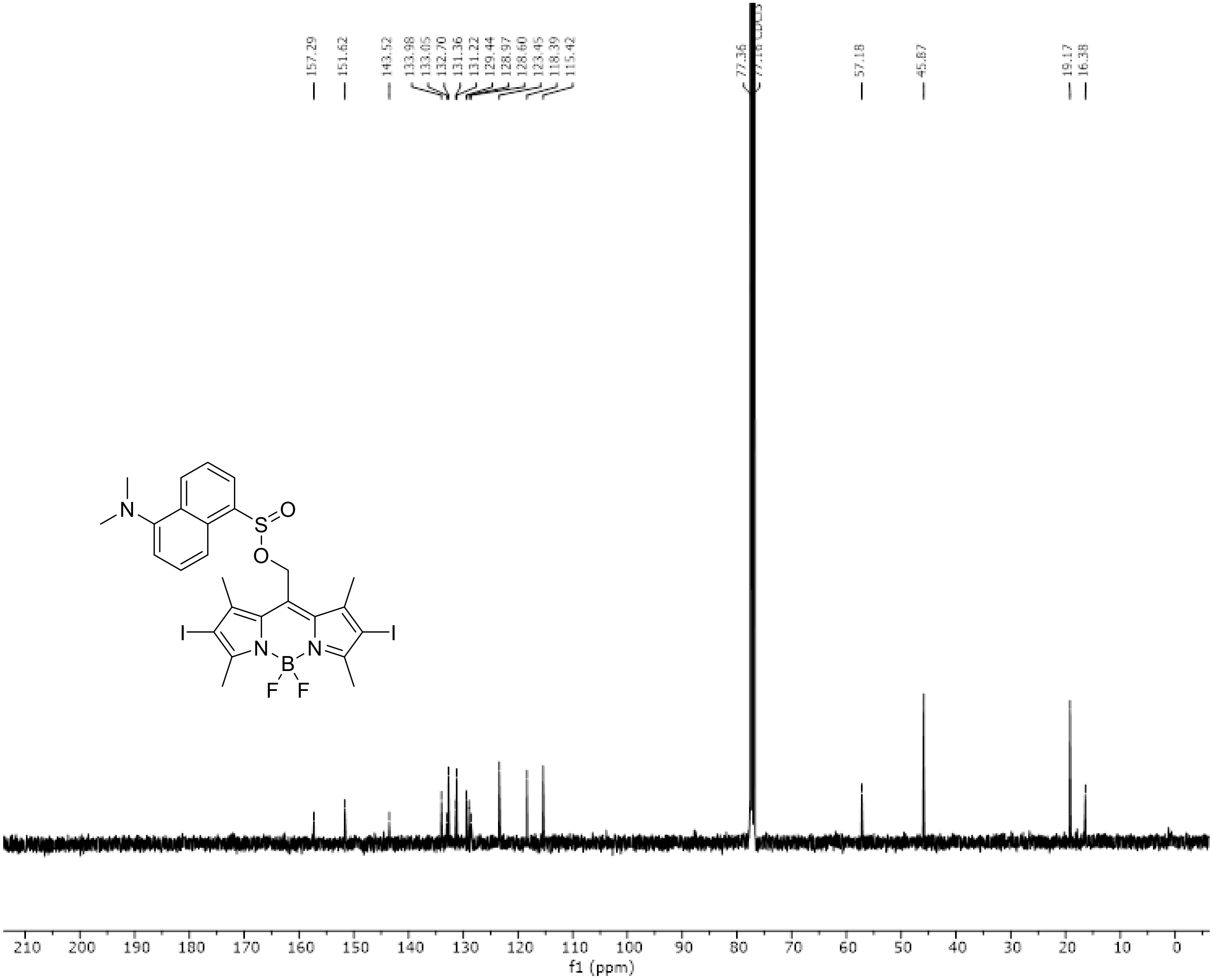

Figure S25. ^{13}C NMR (100.62 Hz) of **4-I** in $CDCl_3$

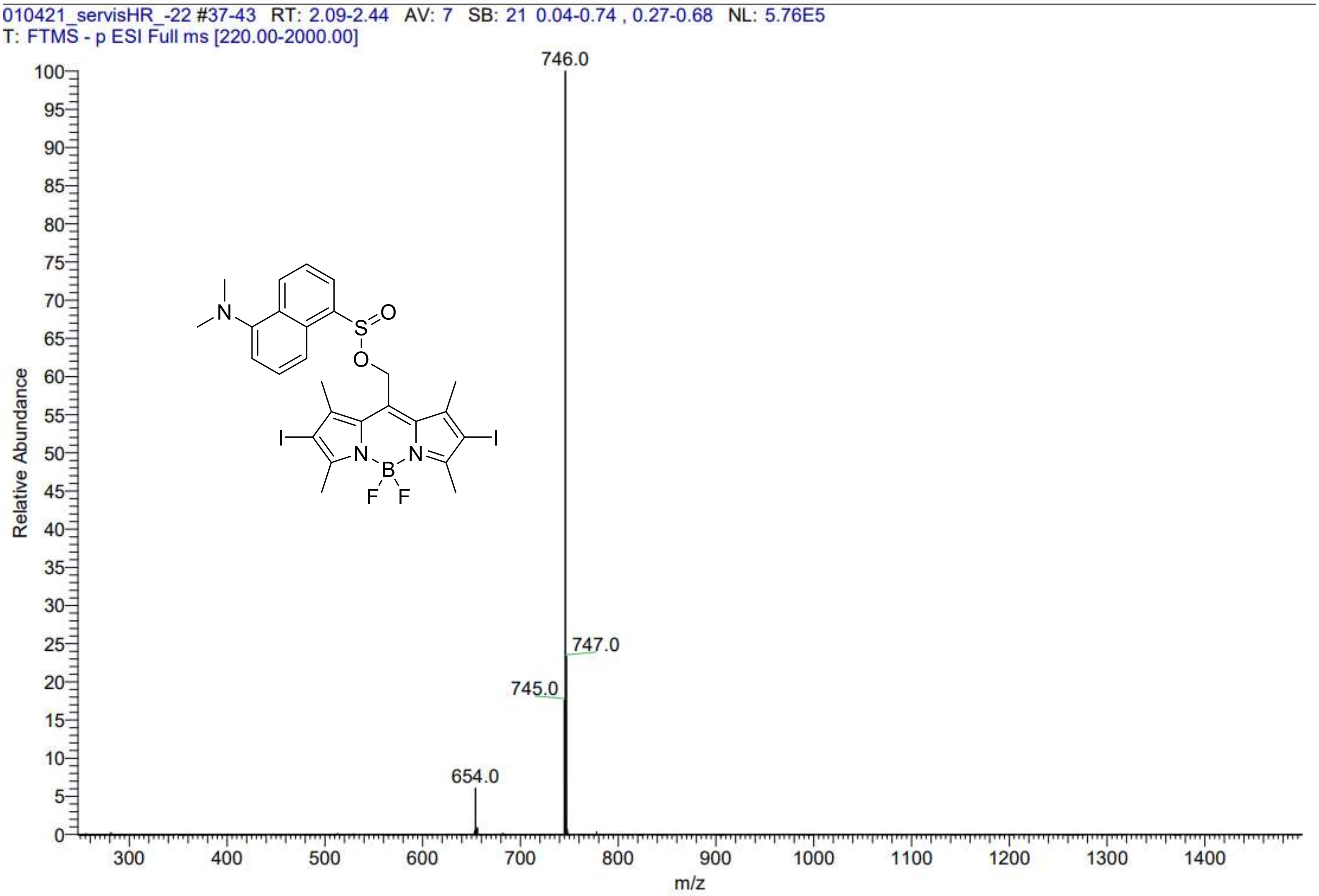

Figure S26. HRMS (ESI-) spectrum of **4-I**

Absorption and emission spectra

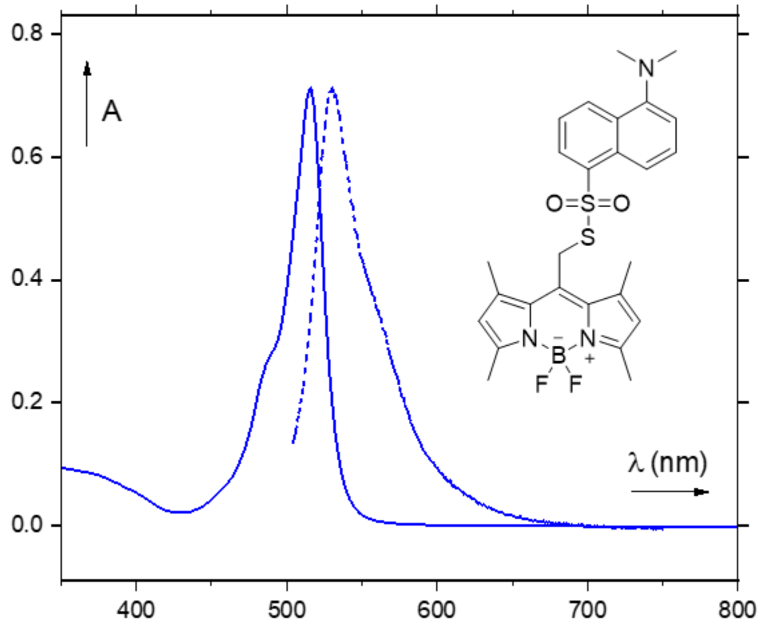

Figure S27. Absorption and emission spectrum of **1**, *c* = 1.5x10^{-5}M, MeOH/DCM (2/8, v/v), excitation wavelength 500 nm

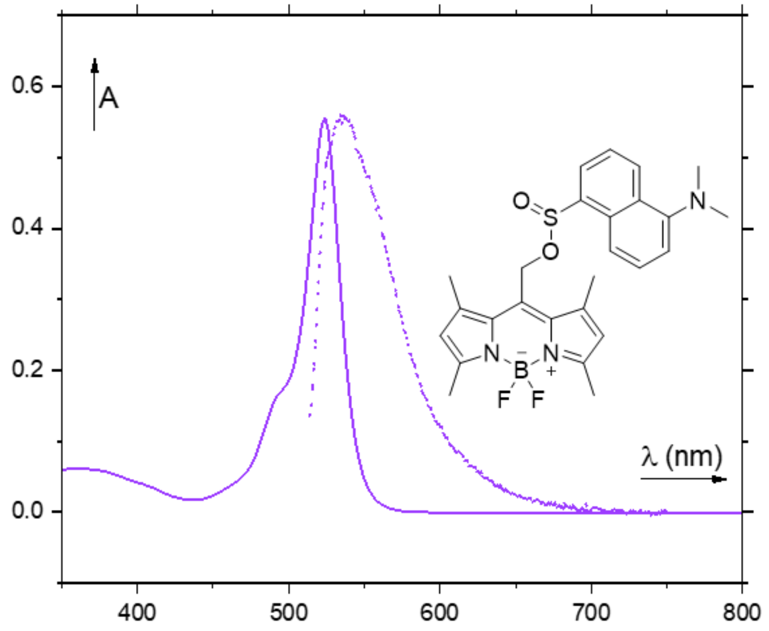

Figure S28. Absorption and emission spectrum of **4-H**, *c* = 1.5x10^{-5}M, MeOH/DCM (2/8, v/v), excitation wavelength 500 nm

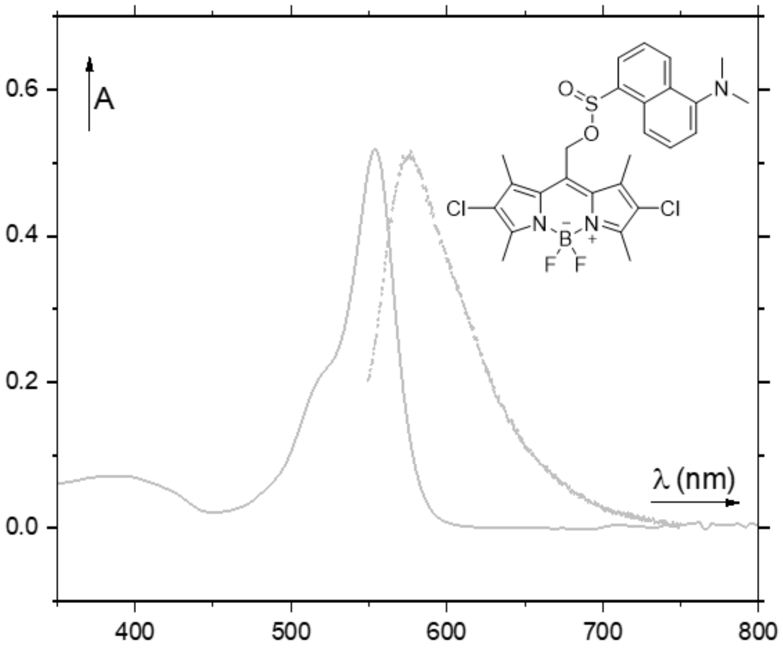

Figure S29. Absorption and emission spectrum of **4-Cl**, *c* = 1.5x10^{-5}M, MeOH/DCM (2/8, v/v), excitation wavelength 545 nm

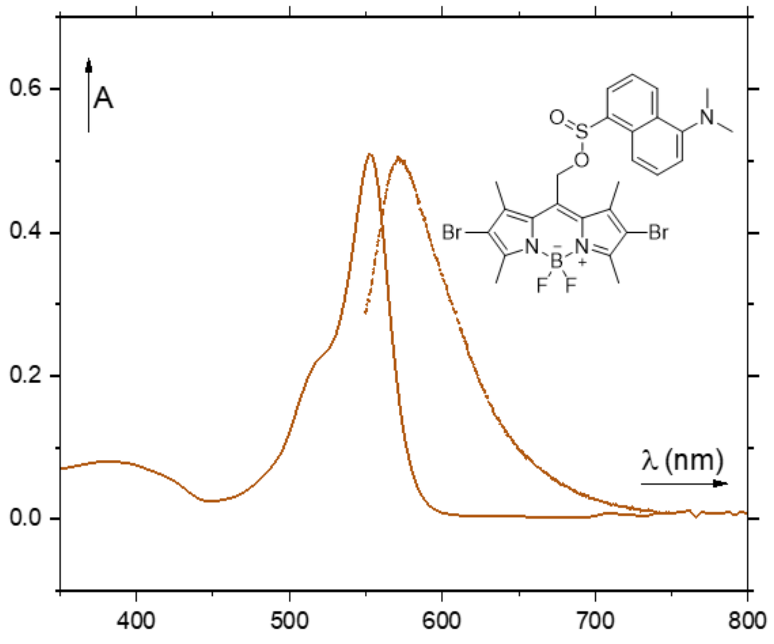

Figure S30. Absorption and emission spectrum of **4-Br**, *c* = 1.5x10^{-5}M, MeOH/DCM (2/8, v/v), excitation wavelength 545 nm

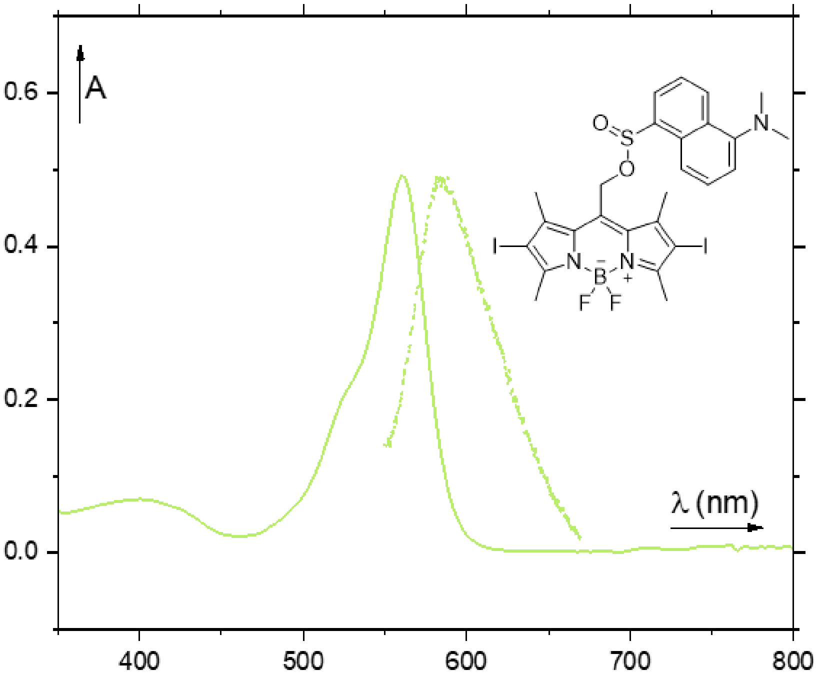

Figure S31. Absorption and emission spectrum of **4-I**, c = 1.5x10^{-5}M, MeOH/DCM (2/8, v/v), excitation wavelength 545 nm

Reactivity in DMSO:water mixture

Compoud **1** was dissolved in DMSO and dilluted in water at a 1:1 ratio. Then, the solution was irradiated with 525 nm LED, and absorption and emission spectra were measured at selected timepoints.

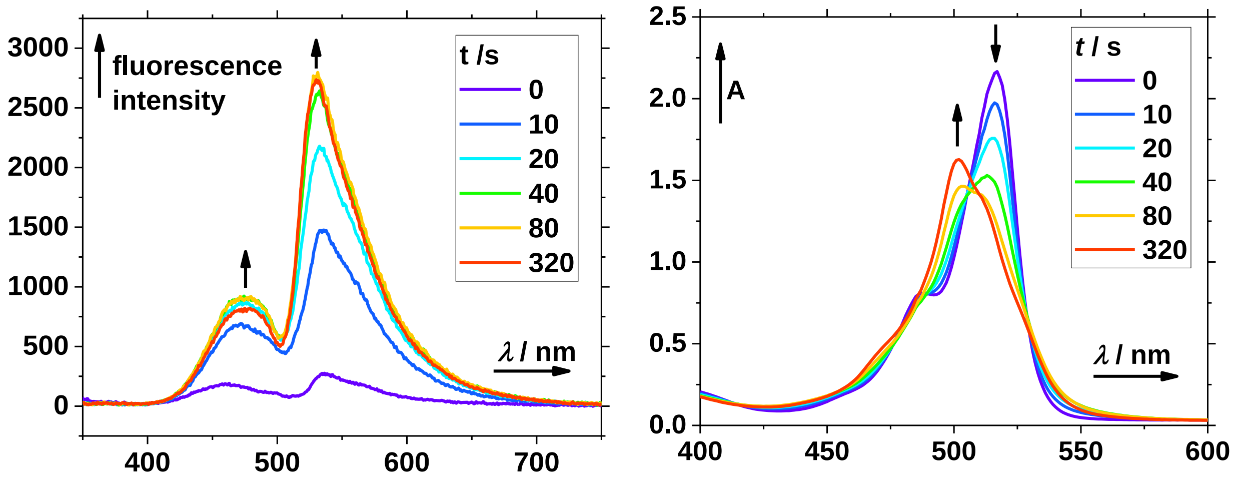

Figure S32. Irradiation of **1** with a 525-nm LED in a non-degassed mixture of DMSO/water (1:1, $c \approx 6 \times 10^{-5}$ M)

Cell viability assay

A HeLa S3 cell line (cat. no. ATCC CCL-2, ATCC, USA) was cultivated in RPMI-1640 medium (cat. no. R7638; Merck, Germany) supplemented with 10% fetal bovine serum (cat. no. F7524, Merck, Germany; inactivated at 56 °C for 30 minutes) and 1% L-glutamine (cat. no. G7513, Merck, Germany). Cells were cultured in an incubator (37 °C, 5% CO_2). Adherent cells were dissociated using a Trypsin-EDTA solution (cat. no. T4049-500 mL, Merck, Germany). The cells were collected, aliquoted into Eppendorf tubes and treated with **1** at concentrations of 1 µM, 10 µM, 100 µM for 5 min. One tube was incubated in the dark, and another tube was irradiated for 5 min with a 525 nm LED. Subsequently, the cells were placed on white 384-well microtitration plates (cat. no. 781981, F-bottom, sterile, BRAND GMBH, Germany) at a concentration of 10 000 cells per well, in octaplicates. According to the manual, cell viability was measured after 6 h and 24 h post treatment with CellTiter-Glo® Luminescent Cell Viability Assay (cat. no. G7570, Promega, USA).

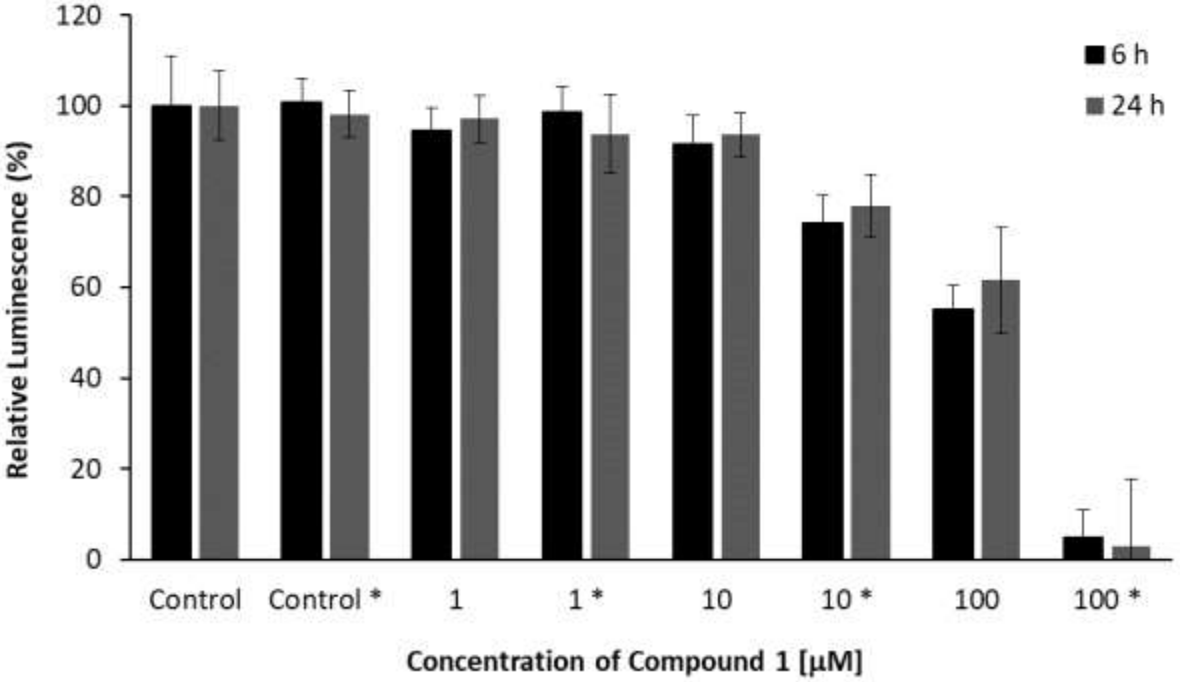

Figure S33. Cell viability of **1**. HeLa S3 cells were exposed to 3 different concentrations of **1** (1 µM, 10 µM, and 100 µM) and irradiated with 525 nm LED (marked as *) for 5 min. Subsequently, cells were placed in 384-well plates, and cell viability was measured after 6 (dark columns) and 24 (grey columns) hours with a CellTiter-Glo® Luminescent Cell Viability Assay. The results are expressed as percentage of relative luminescence normalized to that of the control – untreated cells (mean ± SD, n = 8).

Confocal microscopy

A U-2 OS cell line (cat. no. ACC-785, DSMZ) was cultured in DMEM high glucose 4.5g/L medium (cat. no. R8758, Merck, Germany) supplemented with 10% fetal bovine serum - FBS (cat. no. F7524, Merck, Germany; inactivated at 56 °C for 30 minutes) and 1% L-glutamine (cat. no. G7513, Merck, Germany). Cells were cultured in an incubator (37°C, 5% CO_2). Adherent cells were dissociated using a Trypsin-EDTA solution (cat. no. T4049-500 mL, Merck, Germany) and seeded into 96-well glass bottom plates (Cellvis, P96-1.5H-N) at a concentration of 30 000 cells per well in complete Leibowitz L-15 medium (10% FBS) 24 hours prior to the experiment. Immediately before the experiment, the medium was changed to a complete L-15 medium with 100 µM of **1** and left standing in the dark

for 1 min. Subsequently, the cells were continuously irradiated with a 492-nm laser (40% power) for 3 minutes, setting the detector (GaAsP) range to 516 - 543 nm. The first and last images are shown in the manuscript (Figure 2e-f). As a control, cells were imaged at the beginning and 3 minutes later, in the dark, using the same settings (Figure S34), under a confocal microscope ZEISS LSM 980 fitted with an incubator and a 20x/0.75 Plan-Apochromat objective (Carl Zeiss Jena GmbH, Germany). The images were processed in Zeiss Imaging Software ZEN 3.2 (blue edition) and Fiji software (2.9.0), calculating the mean pixel value of 8-bit images (Figures S35, irradiated sample, and S36, dark control).

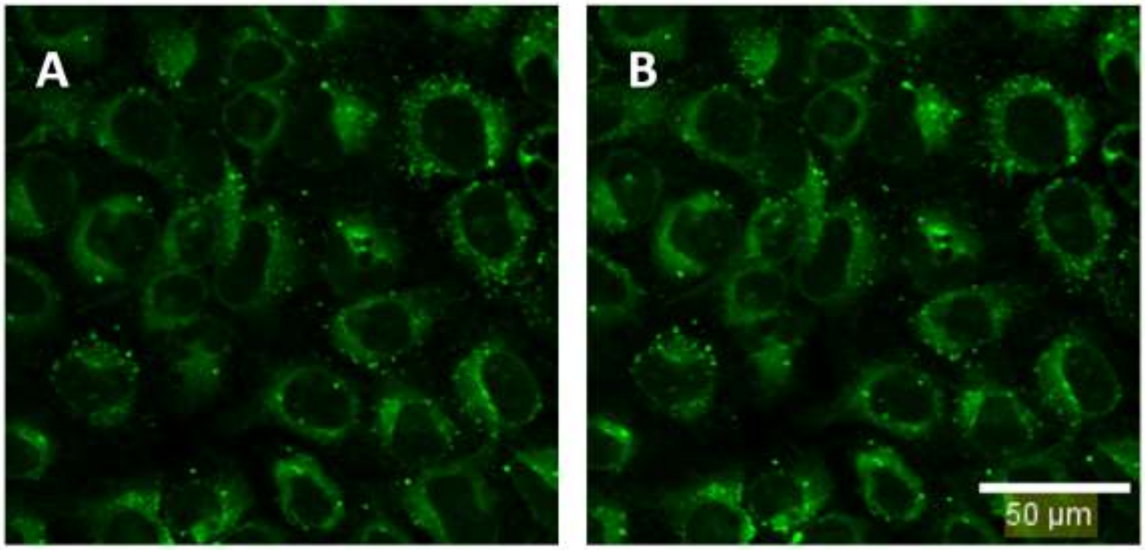

Figure S34. Fluorescence images of U-2 OS cells treated with **1** (c = 100 μM) imaged after 1 (A) and 3 (B) min in the dark. The scale bar is 50 μm.

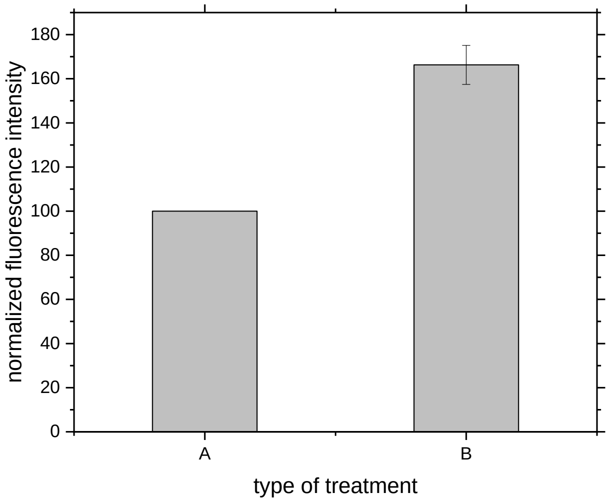

Figure S35. The mean pixel value of 8-bit images from Figure 2e (column A) and 2f (column B) was normalized to the value of column A (the fluorescence intensity of cells after 1 min incubation with **1** before irradiation). The data were collected in triplicates and showed fluorescence increase by a factor of 1.7.

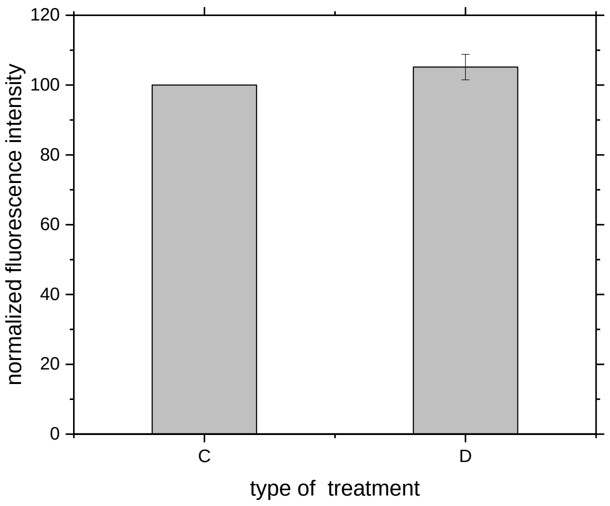

Figure S36. The mean pixel value of 8-bit images from Figure S34A (column C) and S34B (column D) was normalized to the value of column C (the fluorescence intensity of cells after 1 min incubation with **1**). The data were collected in triplicates and showed no fluorescence buildup by standing in the dark (dark control).

HPLC-MS analysis

The reaction in phosphate buffer (PBS, cat. No. D8537, Sigma-Aldrich) was initiated by dissolving **1** in a final concentration of 1 µM (from 1 mM DMSO stock solution) in 1.5 mL Eppendorf tubes. The tubes were irradiated at 525 nm or kept at room temperature in the dark for 5 minutes. The reactions were stopped immediately by adding an equal volume of 100% acetonitrile with 40 mM *N*-ethylmaleimide (NEM) as the derivatization agent[10] and kept in the dark on ice. All experiments were performed in triplicates.

For analysis, 2 µL of the sample was injected into an Agilent 1290 HPLC system and separated on a Luna Omega 1.6 µm Polar C18 Column (100 x 2.1 mm) using a mobile phase consisting of water with 0.1% formic acid (A) and acetonitrile with 0.1% formic acid (B), gradient of 2 to 95% B over 6.5 minutes, and a flow rate of 0.5 mL/min.

The Sciex 6500 triple-quadrupole mass spectrometer, operating with an electrospray ionization source, was run in multiple-reaction-monitoring (MRM) mode, with the interface heated to 350 °C. The declustering potential was 20 V, the entrance potential was 10 V, and the collision energy was 28 eV. The characteristic product ions were (**1**) m/z 528.2 -> 508.1, (**5**) 279.1 -> 197.8, GSSG 613.0 -> 355.1, and GSH-NEM 433.2 -> 304.1, which were used for quantification.

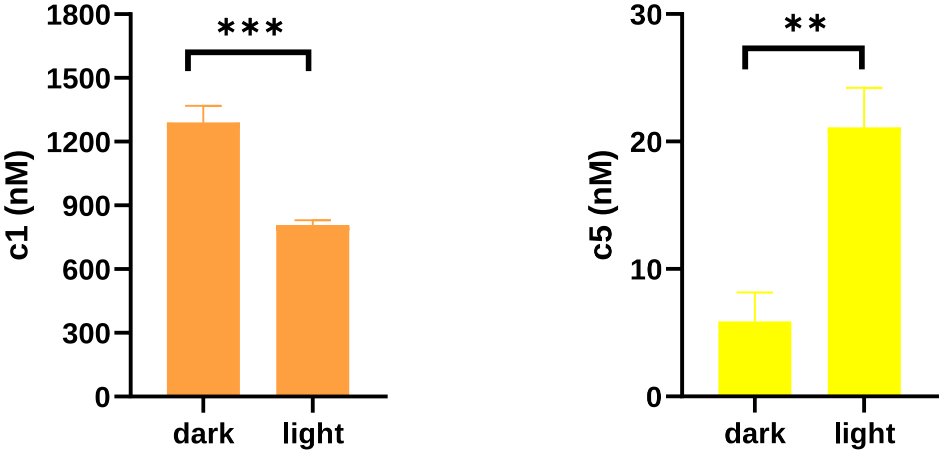

Figure S37. Concentration of **1** (c = 1 µM) before and after irradiation in aqueous soutions determined by LCMS (left) and the corresponding increase of the concentration of **5** in the respective solution (right). Note: The conversion of **1** to **5** remained below 50%. The presence of **5** in the solution before irradiation indicates partial conversion of **1** to **5** during the sample preparation. The quantity of photoproduced **5** decreased in subsequent processes (e.g., photooxidation and bleaching) and did not correspond to the quantity of converted **1**. The results are presented mean ± SD, n = 3. The Student's t test was used for statistics evaluation, two stars indicate P < 0.01, three stars indicate P < 0.001.

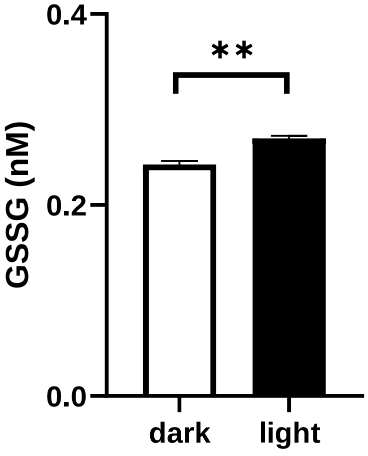

Figure S38. Concentration of GSSG found in the mixture of **1** (c = 1 µM) in the presence of GSH (c = 1 µM) before (left) and after irradiation (right) The results are presented mean ± SD, n = 3. The Student's t test was used for statistics evaluation, two stars indicate P < 0.01.

Suggested mechanism

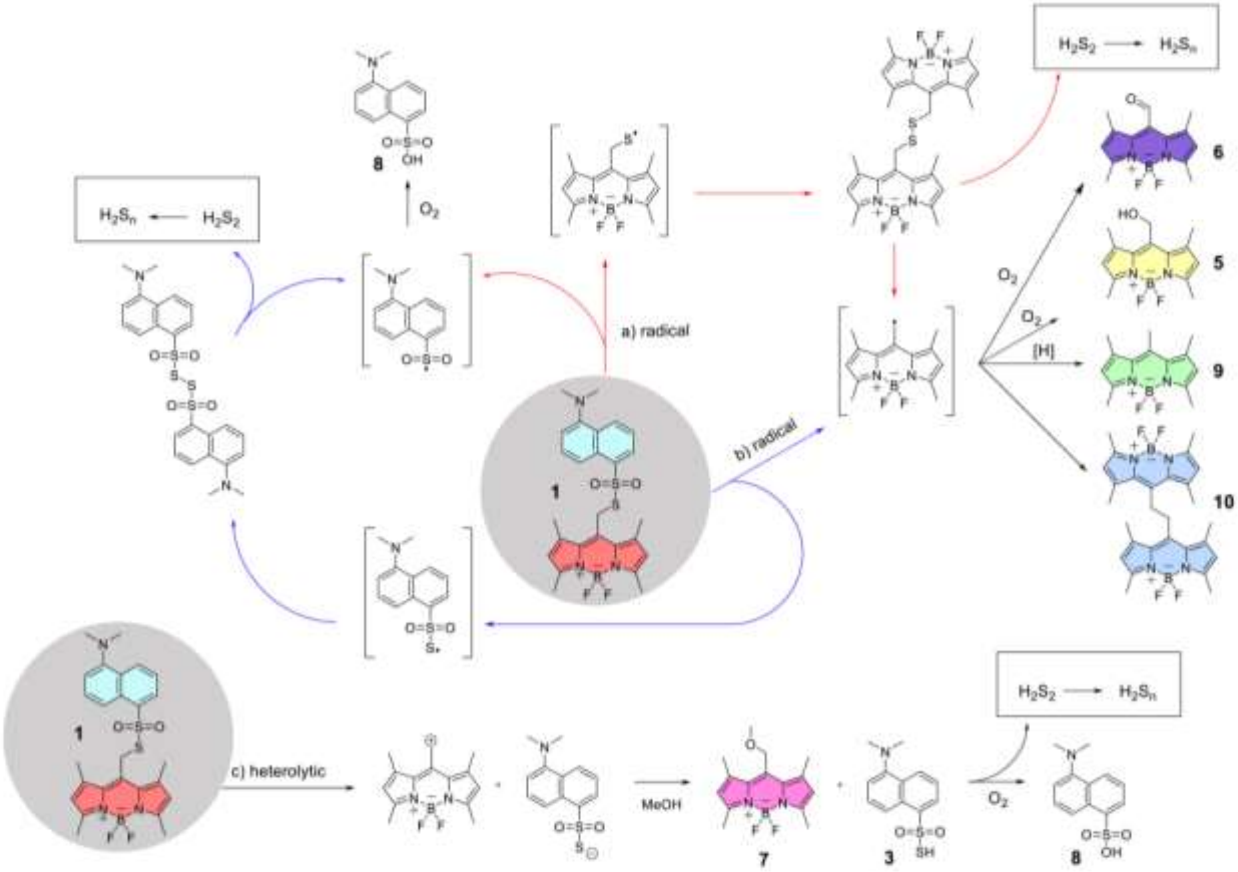

Scheme S4. Suggested Mechanistic Pathways of Photodegradation of **1**

References

(1) Venkateswarulu, M.; Kumar, S.; Ghosh, S. Modified Atomic Orbital Overlap: Molecular Level Proof of the Nucleophilic Cleavage Propensity of Dinitrophenol-Based Probes. *J. Org. Chem.* **2017**, *82* (9), 4713–4720. https://doi.org/10.1021/acs.joc.7b00317.

(2) Slanina, T.; Shrestha, P.; Palao, E.; Kand, D.; Peterson, J. A.; Dutton, A. S.; Rubinstein, N.; Weinstain, R.; Winter, A. H.; Klán, P. In Search of the Perfect Photocage: Structure–Reactivity Relationships in Meso-Methyl BODIPY Photoremovable Protecting Groups. *J. Am. Chem. Soc.* **2017**, *139* (42), 15168–15175. https://doi.org/10.1021/jacs.7b08532.

(3) Banerjee, A.; Sarkar, S.; Shah, J. A.; Frederiks, N. C.; Bazan-Bergamino, E. A.; Johnson, C. J.; Ngai, M.-Y. Excited-State Copper Catalysis for the Synthesis of Heterocycles. *Angewandte Chemie International Edition* **2022**, *61* (4), e202113841. https://doi.org/10.1002/anie.202113841.

(4) Kaufmann, J.; Müller, P.; Andreadou, E.; Heckel, A. Green-Light Activatable BODIPY and Coumarin 5'-Caps for Oligonucleotide Photocaging. *Chemistry – A European Journal* **2022**, *28* (36), e202200477. https://doi.org/10.1002/chem.202200477.

(5) Donnelly, J. L.; Offenbartl-Stiegert, D.; Marín-Beloqui, J. M.; Rizzello, L.; Battaglia, G.; Clarke, T. M.; Howorka, S.; Wilden, J. D. Exploring the Relationship between BODIPY Structure and Spectroscopic Properties to Design Fluorophores for Bioimaging. *Chemistry – A European Journal* **2020**, *26* (4), 863–872. https://doi.org/10.1002/chem.201904164.

(6) Reinfelds, M.; Hermanns, V.; Halbritter, T.; Wachtveitl, J.; Braun, M.; Slanina, T.; Heckel, A. A Robust, Broadly Absorbing Fulgide Derivative as a Universal Chemical Actinometer for the UV to NIR Region. *ChemPhotoChem* **2019**, *3* (6), 441–449. https://doi.org/10.1002/cptc.201900010.

(7) Cline, J. D. Spectrophotometric Determination of Hydrogen Sulfide in Natural Waters1. *Limnology and Oceanography* **1969**, *14* (3), 454–458. https://doi.org/10.4319/lo.1969.14.3.0454.

(8) Zhang, M.; Ding, X.; Lu, A.; Kang, J.; Gao, Y.; Wang, Z.; Li, H.; Wang, Q. Generation and Precise Control of Sulfonyl Radicals: Visible-Light-Activated Redox-Neutral Formation of Sulfonates and Sulfonamides. *Org. Chem. Front.* **2021**, *8* (5), 961–967. https://doi.org/10.1039/D0QO01413C.

(9) Liu, C.; Chen, W.; Shi, W.; Peng, B.; Zhao, Y.; Ma, H.; Xian, M. Rational Design and Bioimaging Applications of Highly Selective Fluorescence Probes for Hydrogen Polysulfides. *J. Am. Chem. Soc.* **2014**, *136* (20), 7257–7260. https://doi.org/10.1021/ja502968x.

(10) Giustarini, D.; Dalle-Donne, I.; Milzani, A.; Fanti, P.; Rossi, R. Analysis of GSH and GSSG after Derivatization with N-Ethylmaleimide. *Nat Protoc* **2013**, *8* (9), 1660–1669. https://doi.org/10.1038/nprot.2013.095.
